# Enhanced NH_3_ Synthesis from Air in a Plasma Tandem-Electrocatalysis System Using Plasma-Engraved N-Doped Defective MoS_2_

**DOI:** 10.1021/jacsau.3c00087

**Published:** 2023-04-26

**Authors:** Jiageng Zheng, Hao Zhang, Jiabao Lv, Meng Zhang, Jieying Wan, Nick Gerrits, Angjian Wu, Bingru Lan, Weitao Wang, Shuangyin Wang, Xin Tu, Annemie Bogaerts, Xiaodong Li

**Affiliations:** † State Key Laboratory of Clean Energy Utilization, College of Energy and Engineering, Academy of Ecological Civilization, Zhejiang University, Hangzhou 310027, China; ‡ College of Optical Science and Engineering, Zhejiang University, Hangzhou 310027, China; § Research Group PLASMANT, Department of Chemistry, University of Antwerp, Universiteitsplein 1, BE-2610 Wilrijk, Belgium; ∥ Department of Electrical Engineering and Electronics, University of Liverpool, Liverpool L69 3GJ, U.K.; ⊥ State Key Laboratory of Chem/Bio-Sensing and Chemometrics, College of Chemistry and Chemical Engineering, Hunan University, Changsha 410082, China

**Keywords:** sustainable NH_3_ production, plasma electrocatalysis, defective N-MoS_2_, plasma engraving, density functional theory

## Abstract

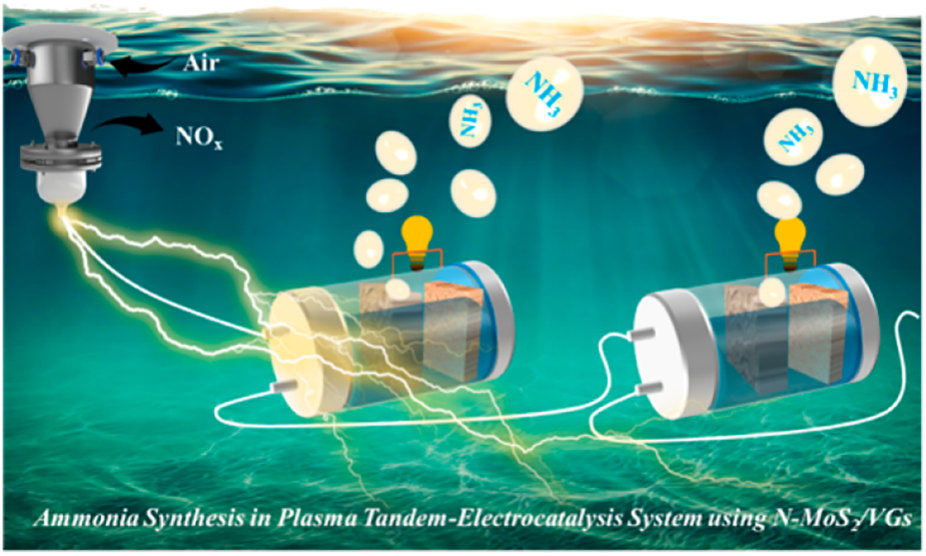

We have developed a sustainable method to produce NH_3_ directly from air using a plasma tandem-electrocatalysis system that operates via the N_2_–NO_*x*_–NH_3_ pathway. To efficiently reduce NO_2_^–^ to NH_3_, we propose a novel electrocatalyst consisting of defective N-doped molybdenum sulfide nanosheets on vertical graphene arrays (N-MoS_2_/VGs). We used a plasma engraving process to form the metallic 1T phase, N doping, and S vacancies in the electrocatalyst simultaneously. Our system exhibited a remarkable NH_3_ production rate of 7.3 mg h^–1^ cm^–2^ at −0.53 V vs RHE, which is almost 100 times higher than the state-of-the-art electrochemical nitrogen reduction reaction and more than double that of other hybrid systems. Moreover, a low energy consumption of only 2.4 MJ mol_NH_3__^–1^ was achieved in this study. Density functional theory calculations revealed that S vacancies and doped N atoms play a dominant role in the selective reduction of NO_2_^–^ to NH_3_. This study opens up new avenues for efficient NH_3_ production using cascade systems.

## Introduction

Ammonia (NH_3_) is essential for modern agriculture^1,2^ and is being explored as a promising carbon-free hydrogen carrier to tackle climate change and energy challenges.^3^ However, the conventional centralized Haber–Bosch process for industrial NH_3_ production requires extremely harsh conditions,^4^ resulting in high energy demands^5^ and significant CO_2_ emissions.^6^ Therefore, developing sustainable, environmentally friendly, and distributable routes for NH_3_ production is critical.

One promising approach is electrochemical nitrogen reduction reaction (eNRR) to NH_3_, which can be powered by renewable electricity using abundant N_2_/air and H_2_O under ambient conditions.^7−11^ The performance of electrocatalysts is crucial for eNRR kinetics. Among the noble-metal-based,^12,13^ transition-metal-based,^14,15^ and metal-free electrocatalysts^16,17^ explored, Mo, especially MoS_2_, is of particular interest due to its natural occurrence in biological nitrogen fixation and structural similarity to nitrogenase.^18−21^ Recently, there has been a surge in efforts to enhance catalyst reactivity through phase transformation,^22^ heteroatom doping,^23^ and defect engineering.^24^ Nevertheless, the ultrastrong N≡N bond,^25−27^ competitive hydrogen evolution reaction (HER),^28^ and poor solubility of N_2_ in the electrolyte^29^ have limited the NH_3_ production rate to <0.1 mg h^–1^ cm^–2^.

To address these challenges, a promising two-step strategy has been proposed that uses plasma to activate inert N_2_ into more accessible and clean nitrogen oxides (NO_*x*_), followed by enhanced electroreduction of NO_*x*_^–^ into NH_3_.^30−32^ Although NO_*x*_^–^ may be abundant in wastewater, it would be unsuitable for this application due to the presence of impurities. The plasma electrocatalysis approach uses air instead of pure nitrogen, eliminating the need for energy-intensive air separation and purification processes, and is suitable for intermittent and decentralized operation using renewable energy sources as a turnkey process.^33−35^ While an NH_3_ production rate of up to 3.0 mg h^–1^ cm^–2^ has been achieved,^36,37^ investigations into this strategy remain extremely limited. Further development of new electrochemical NO_*x*_ reduction reaction (eNO_*x*_^–^RR) processes, including system design and high-performance catalysts such as MoS_2_, is critical to improve the slow conversion of NO_*x*_^–^ to NH_3_ in the second step. Moreover, existing catalyst engineering strategies typically require high temperature (>300 °C)^38^ and long-duration treatment (>2 h),^24^ calling for a more facile and rational catalyst fabrication approach. Furthermore, a molecular-level understanding of the complex eNO_*x*_^–^RR mechanism remains limited, requiring in-depth experimental and theoretical studies.

Herein we propose a plasma tandem-electrocatalysis system to generate NH_3_ from air (see Plasma Tandem-Electrocatalysis Setup and Figure S1). We engineered a novel defective N-MoS_2_ electrocatalyst supported by vertical graphene arrays on carbon cloth (VGs/CC) with exceptional eNO_*x*_^–^RR reactivity. Advanced physical characterizations and density functional theory (DFT) calculations were conducted to elucidate the eNO_*x*_^–^RR reaction pathways with N-MoS_2_/VGs.

## Results and Discussion

**Figure 1:**
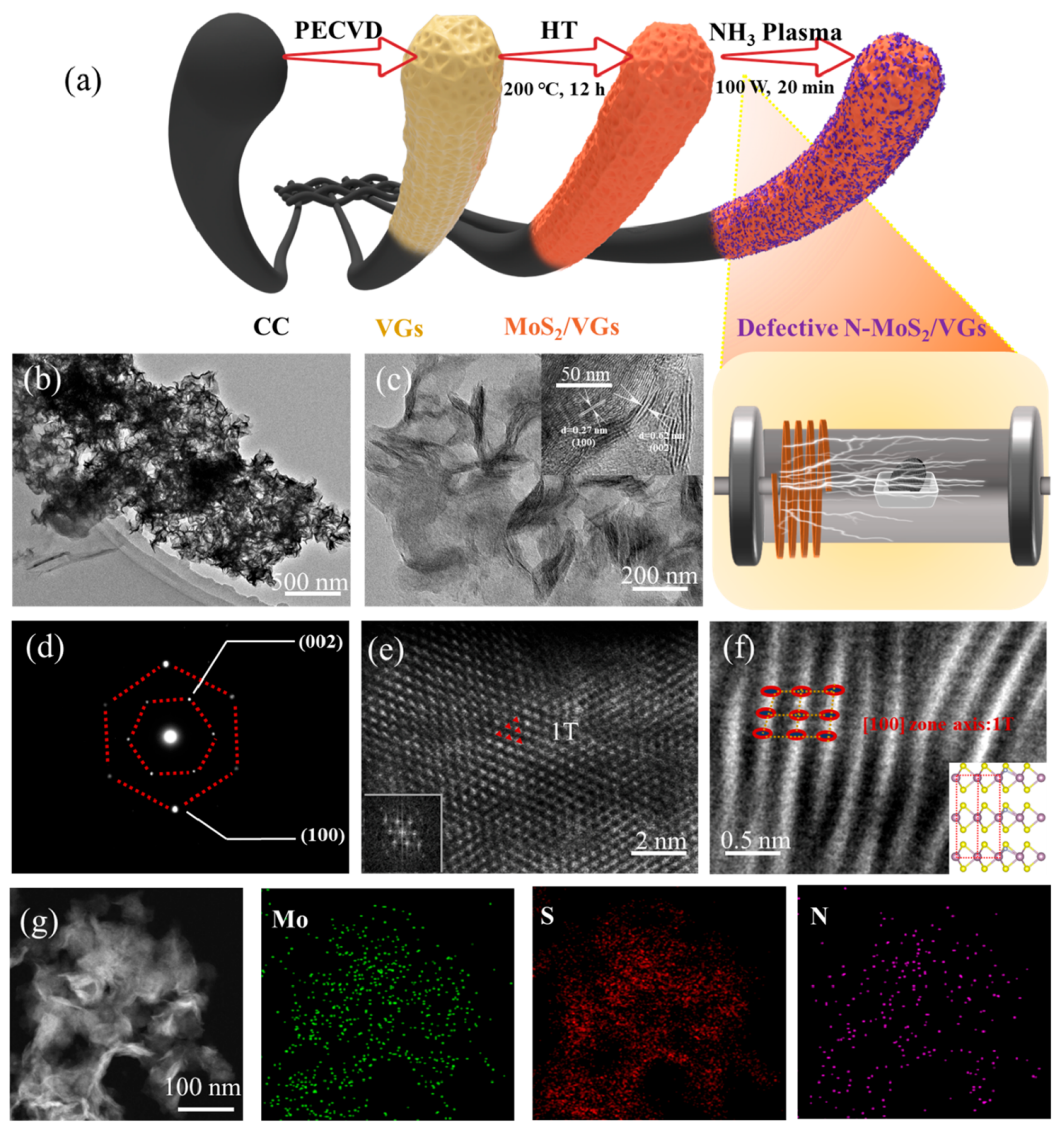
(a) Schematic illustration for N-MoS_2_/VGs fabrication procedure and RF NH_3_ plasma engraving process. (b) TEM image, (c) HRTEM image, (d) SAED pattern, (e) HAADF image, (f) AC-STEM image, and (g) EDX mapping of N-MoS_2_/VGs.

The MoS_2_ nanosheets were assembled by a hydrothermal (HT) method and supported by vertical graphene arrays obtained through plasma-enhanced chemical vapor deposition (PECVD) on CC (Figure 1a and Catalyst Synthesis). The transmission electron microscopy (TEM) image (Figure 1b) confirmed the formation of a well-defined nanosheet profile. The marked space lattice fringe and distinct spots in the high-resolution transmission electron microscopy (HRTEM) image (Figure 1c) together with the selected-area electron diffraction (SAED) pattern (Figure 1d) revealed the (002) and (100) planes of MoS_2_.

Notably, we pioneered a radiofrequency (RF) NH_3_ plasma strategy for atomic-level surface engineering of MoS_2_ (Figure 1a). By creation of a plasma sheath on the sample surface, ablated Mo atoms reacted with N atoms in the plasma to form N-MoS_2_. Furthermore, the plasma treatment also generated crystal vacancies via ion sputtering and etching effects of NH_3_.^39−41^ This method required only a short duration (20 min) and low temperature (25 °C). The formation of octahedrally coordinated 1T-MoS_2_ after the plasma engraving was confirmed by high-angle annular dark-field (HAADF) (Figure 1e) and spherical aberration corrected scanning transmission electron microscopy (AC-STEM) along the (100) zone axis (Figure 1f). The HRTEM images of MoS_2_/VGs and N-MoS_2_/VGs further evidenced the distinct phase transformation from 2H to 1T (Figures S2 and S3). Additionally, energy-dispersive X-ray (EDX) elemental mapping images demonstrated the uniform distribution of Mo, S, and N in the N-MoS_2_/VGs (Figure 1g).

**Figure 2:**
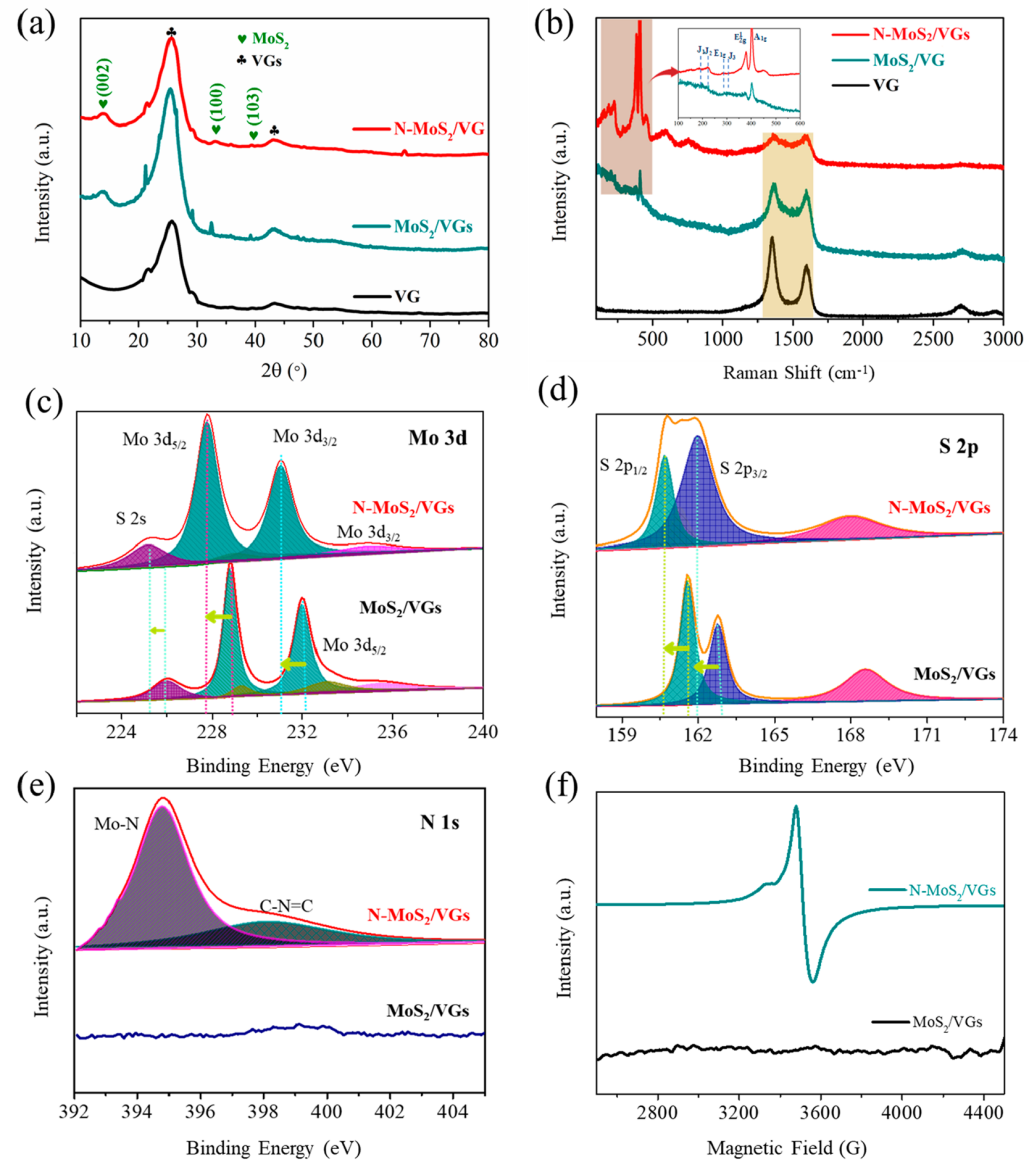
(a) XRD patterns and (b) Raman spectra of N-MoS_2_/VGs, MoS_2_/VGs, and VGs. (c–e) XPS spectra of (c) Mo 3d, (d) S 2p, and (e) N 1s over N-MoS_2_/VGs and MoS_2_/VGs. (f) EPR spectra of N-MoS_2_/VGs and MoS_2_/VGs.

X-ray diffraction (XRD) patterns (Figure 2a) reveal characteristic peaks at 14.37°, 32.68°, and 39.54° that correspond to the (002), (100), and (103) planes of MoS_2_ (PDF no. 37-1492) for N-MoS_2_/VGs, MoS_2_/VGs, and VGs. The 1T phase of N-MoS_2_/VGs is further confirmed by Raman spectroscopy (Figure 2b). The E_2g_ and A_1g_ peaks at 380 and 405 cm^–1^, respectively, are observed in both MoS_2_/VGs and N-MoS_2_/VGs, with N-MoS_2_/VGs also exhibiting three additional peaks (148, 207, and 335 cm^–1^) associated with vibrational modes of the 1T phase. The E_2g_ peak (380 cm^–1^) is also found in N-MoS_2_/VGs due to the well-organized tetragonal symmetry.^42−44^ X-ray photoelectron spectroscopy (XPS) (Figure 2c–e) revealed the presence of Mo, S, and N in N-MoS_2_/VGs and the absence of N in MoS_2_/VGs. The Mo 3d spectrum of MoS_2_/VGs can be deconvoluted into four peaks, with two dominant peaks at 232.0 eV (Mo 3d_3/2_) and 229.1 eV (Mo 3d_5/2_) assigned to the 1T phase and two other peaks at 233.5 eV (Mo 3d_3/2_) and 229.6 eV (Mo 3d_5/2_) allocated to the 2H phase.^45,46^ Conversely, only two typical 1T phase peaks at 231.2 eV (Mo 3d_3/2_) and 229.1 eV (Mo 3d_5/2_) are detected in N-MoS_2_/VGs. Furthermore, the positive shift of N-MoS_2_/VGs to MoS_2_/VGs also confirms the phase transformation from 2H to 1T. Notably, the S:Mo ratio decreased from 1.74 to 1.37, according to quantized EDX data (Table S1), likely due to the substitution of N and possible S vacancies induced by plasma engraving. This hypothesis is explicitly proven by the electron paramagnetic resonance (EPR) spectroscopy results (Figure 2f), which display a prominent signal at *g* = 2.004 assigned to S vacancies in N-MoS_2_/VGs and no signal in MoS_2_/VGs, suggesting the partial deletion of thiol–Mo bonds in N-MoS_2_/VGs.^47^

**Figure 3:**
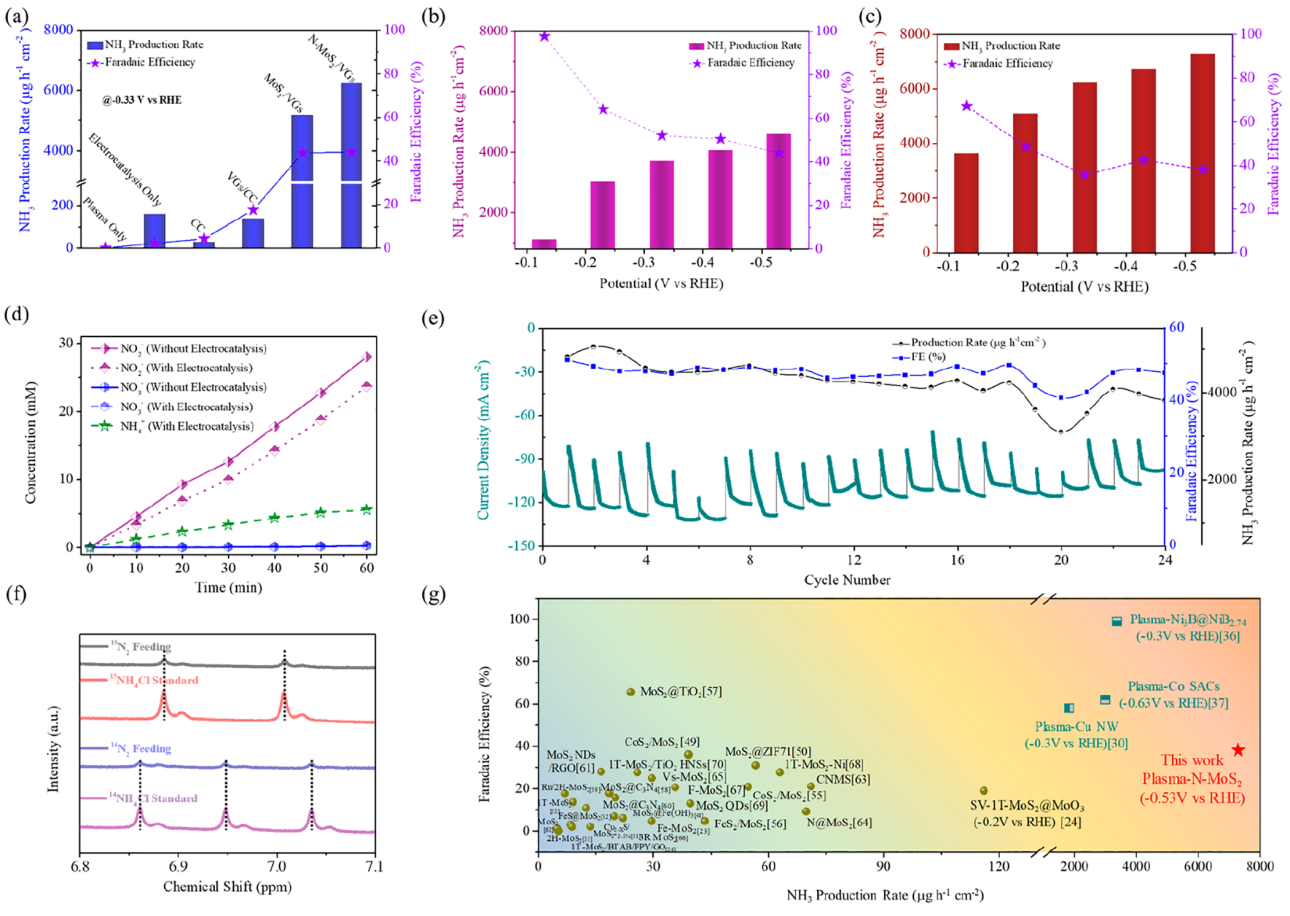
(a–c) NH_3_ production rates and FEs for (a) plasma only, N-MoS_2_/VGs electrocatalysis only, and plasma electrocatalysis with different catalysts, (b) plasma single-electrocatalysis, and (c) plasma tandem-electrocatalysis. (d) Time-dependent concentrations of nitrate, nitrite, and NH_4_^+^ with/without electrocatalysis in the hybrid system. (e) Stability tests. (f) ^1^H NMR (600 MHz) spectra in ^14^N_2_ or ^15^N_2_ atmosphere after N-MoS_2_/VGs electrocatalysis. (g) Comparison of eNRR using different MoS_2_ catalysts (green symbols), other plasma-electrocatalysis systems (indigo symbols), and this work (red star). See Tables S6 and S7 for more details.

Comparative experiments in the plasma or electrocatalysis alone system and the plasma electrocatalysis system with different catalysts were performed at −0.33 V versus reversible hydrogen electrode (V vs RHE). As shown in Figure 3a, neither the plasma- or electrocatalysis-alone system nor the eNO_*x*_^–^RR over the substrates (CC and VGs/CC) yielded significant NH_3_. However, the MoS_2_ catalyst dramatically enhanced the NH_3_ production rate (5.2 mg h^–1^ cm^–2^) by a factor of approximately 38 over the substrates. Notably, the defective N-MoS_2_/VGs catalyst further improved the catalytic reactivity by over 20%, reaching a production rate of up to 6.2 mg h^–1^ cm^–2^. This enhancement is attributed to the improved electron conductivity, higher charge polarization, and increased active sites of N-MoS_2_/VGs, as discussed above.

In this study, plasma tandem-electrocatalysis was utilized by coupling double electrocatalyst cells, and for comparison, a plasma single-electrocatalysis system was also tested. The results, depicted in Figure 3b,c, demonstrated that increasing potentials had a positive effect on the average NH_3_ production rate but had a negative impact on the Faradaic efficiency (FE) in both modes. A maximum production rate of 4.6 mg h^–1^ cm^–2^ (FE = 44%) and a highest FE of 97.6% (NH_3_ production = 1.12 mg h^–1^ cm^–2^) were achieved, respectively, at −0.53 and −0.13 V vs RHE in the single-electrocatalysis mode. The tandem-electrocatalysis system significantly increased the production rate to 7.3 mg h^–1^ cm^–2^ (FE = 38.1%) at −0.53 V vs RHE (Figure 3c) (see Figures S6–S10 for more comparative results).

The air plasma produced a substantial NO_*x*_ concentration (up to 1.87% with NO:NO_2_ ≈ 5:1) for subsequent eNO_*x*_^–^RR process. To elucidate the origin of NH_3_ in eNO_*x*_^–^RR, we measured the concentrations of NO_2_^–^, NO_3_^–^, and NH_4_^+^ in the electrolyte during plasma discharge with and without electrocatalysis. NO_2_^–^ was found to be the dominant NO_*x*_^–^ (Figure 3d) with a formation rate of 28 mM h^–1^, and it was significantly consumed in the presence of electrocatalysis, strongly indicating that NH_3_ is primarily produced from NO_2_^–^ (referred to as eNO_2_^–^RR hereafter).

The catalyst stability was validated by successive repetitive (24 times in 24 h) tests at −0.33 V vs RHE (Figure 3e). The scanning electron microscopy (SEM) images of the N-MoS_2_/VGs catalyst before and after stability tests revealed no noticeable morphological changes (Figure S13). To confirm the origin of nitrogen for the produced NH_3_, we conducted an isotopic labeling experiment using ^15^N_2_. Consistent with the standard sample, the spectra in Figure 3f exhibited two and three characteristic peaks indexed to ^15^NH_4_^+^ and ^14^NH_4_^+^. Moreover, their production rates determined by NMR spectroscopy exhibited insignificant differences (Figure S14), excluding the influence of the surroundings. Remarkably, the system achieved a maximum NH_3_ production rate of 7.3 mg h^–1^ cm^–2^, which is almost 100 times higher than that of state-of-the-art MoS_2_ eNRR^22−24,48−70^ and over 2 times better than that of other hybrid systems (Figure 3g).^30,36,37^

**Figure 4:**
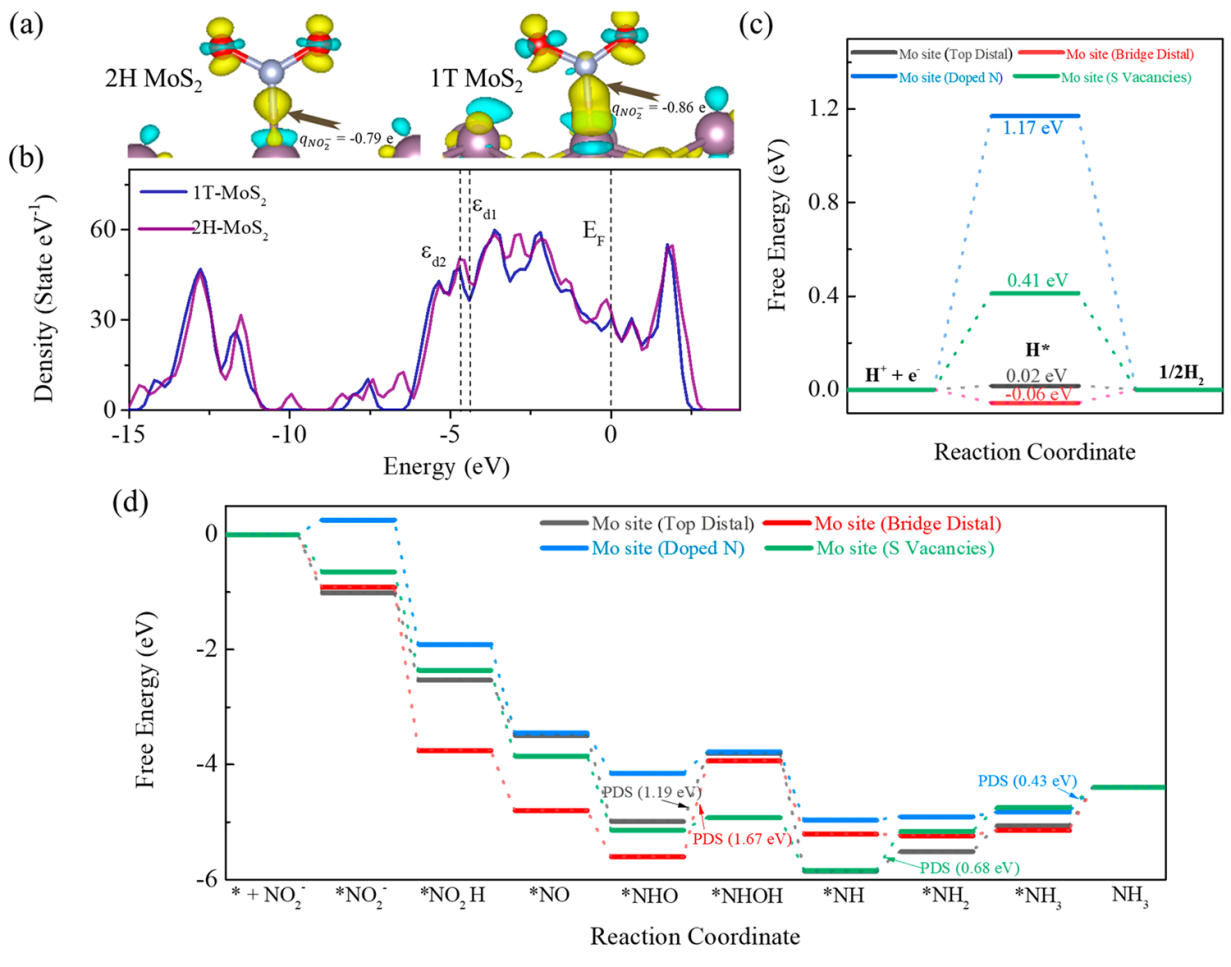
(a) Differential charge densities of adsorbed NO_2_^–^ on 1T-MoS_2_ and 2H-MoS_2_. (b) DOS plots of 1T-MoS_2_ and 2H-MoS_2_. (c) HER and (d) NRR Gibbs free energy diagrams on various adsorption sites.

Moreover, we conducted DFT calculations to elucidate the enhanced eNO_2_^–^RR kinetics of the N-MoS_2_ system (see Section S-VII-1 in the Supporting Information (SI) for details). Our simulation model (Figure S15) featured N-MoS_2_(100) with S vacancies, which correlated with the catalyst characterization results. Figures 4a, S16, and S17 illustrate significant differences in the differential charge density of adsorbed NO_2_^–^ on 2H and 1T phases of MoS_2_. In the 1T phase, the electron accumulation (yellow areas) and electron loss (blue areas) are more extensive, reflecting greater electron delocalization. Bader charge calculations^71^ revealed that NO_2_^–^ chemisorption is more favorable on the metastable 1T-MoS_2_, with an average charge transfer of −0.86*e*, compared to −0.79*e* on 2H-MoS_2_. Furthermore, Figure 4b compares the densities of states (DOS) of 1T-MoS_2_ and 2H-MoS_2_, showing that the d-band center position of 1T-MoS_2_ (ε_d2_ = −3.99 eV) is closer to the Fermi level (*E*_F_) than that of 2H-MoS_2_ (ε_d2_ = −4.17 eV), which favors NO_2_^–^ adsorption. The partial density of states also indicates that 1T-MoS_2_ has a larger overlap of the Mo d valence orbitals with the N p valence orbitals than 2H-MoS_2_ (Figure S18), suggesting that 1T-MoS_2_ has more occupied Mo orbitals and a stronger binding with N atoms from adsorbates such as NO_2_^–^.

We also investigated various adsorption sites of NO_2_^–^ and H^+^ on N-MoS_2_, including Mo top distal (TD), Mo–Mo bridge distal (BD), Mo with N-doped sites (ND), and Mo with S vacancy sites (SV) (Figure S19). We found that eNO_2_^–^RR is inhibited on VGs due to the extremely high adsorption energy (1.54 eV; Table S3). As shown in the Gibbs free energy diagrams of HER in Figure 4c, compared to the adsorption sites for pristine MoS_2_ (Δ*G*_BD_^*^H = −0.06 eV and Δ*G*_TD_^*^H = 0.02 eV), N-doped sites and S vacancy sites in N-MoS_2_ (Δ*G*_ND_^*^H = 1.17 eV and Δ*G*_SV_^*^H = 0.41 eV) yield higher energy barriers, confirming that the enhanced eNO_2_^–^RR selectivity correlates with the suppression of HER. Importantly, the free energy changes of the eNO_2_^–^RR pathways at different adsorption sites (Figure 4d; see Figures S21–S25 for more details) indicate that the energy barrier of the potential-determining step (PDS) for eNO_2_^–^RR in N-doped MoS_2_ (Δ*G*_ND_^PDS^ = 0.43 eV and Δ*G*_SV_^PDS^ = 0.68 eV) exhibits significantly better eNO_2_^–^RR preference than MoS_2_ (Δ*G*_TD_^PDS^ = 1.19 eV and Δ*G*_BD_^PDS^ = 1.67 eV). These findings lead us to conclude that 1T-MoS_2_ facilitates stronger binding and faster electron transfer and that Mo with N doping and S vacancies is beneficial for NH_3_ synthesis.

The optimal energy cost of the system is 1.41 MJ mol_NH_3__^–1^ for plasma NO_*x*_ generation and 0.99 MJ mol_NH_3__^–1^ for eNO_2_^–^RR, yielding a total energy cost of 2.4 MJ mol_NH_3__^–1^. This corresponds to a total of 39.2 kWh kg_NH_3__^–1^, which is at least 24.5% lower than that of the state-of-the-art plasma electrocatalysis systems (Table S7). Moreover, the proposed system has the potential to be extended to a cascade system by coupling plasma with multistage electrocatalyst cells, which can be powered flexibly by intermittent renewable electricity (Tables S8 and S9).

## Conclusions

In summary, we have developed a plasma tandem-electrocatalysis system for NH_3_ synthesis directly from air, achieving an impressive NH_3_ production rate of 7.3 mg h^–1^ cm^–2^ at −0.53 V vs RHE. We engineered a novel defective N-MoS_2_/VGs electrocatalyst, which incorporates metallic 1T-MoS_2_ phase, N doping, and S vacancies through a one-step plasma engraving process. Experimental and DFT investigations confirmed the crucial role of plasma engraving in catalyst fabrication. The adsorption sites of Mo with N doping and S vacancies possess a lower energy barrier for PDS for NH_3_ formation than for H_2_ generation, explaining the enhanced eNO_2_^–^RR performance. This system provides a carbon-free and sustainable approach to NH_3_ synthesis with an enhanced production rate. It also serves as a promising solution for chemical storage of carbon-neutral fuels with high location and scale flexibility, adapting readily to the irregular supply of renewable electricity.

## Experimental Section

### Plasma Tandem-Electrocatalysis Setup

The plasma tandem-electrocatalysis system used in this study is schematically shown in Figure S1. A small-scale atmospheric gliding arc plasma (GAP) reactor was designed to generate NO_*x*_ from air. The reactor consisted of a stainless steel inner anode rod with a diameter of 4 mm and a length of 58 mm and an outer cathode shell (grounded) with a convergent nozzle having a diameter of 21 mm and a thickness of 7.5 mm. The plasma discharge was powered by an AC high voltage power supply (GK-10020L). Dry air was fed into the reactor at a flow rate of 9 L min^–1^, which was controlled by a mass flow controller (MFC, TJ-700C). The arc was ignited at the narrowest gap (kept at 1 mm). The GAP reactor had three radial gas inlets to create more intense flow disturbances inside the reactor, allowing for the formation of an extended plasma area. The reactive plasma system effectively activated the nitrogen bond, leading to the formation of NO_*x*_. To quantify the NO_*x*_ produced with high concentration (NO and NO_2_), the outlet gas was diluted with pure nitrogen and measured using a gas analyzer (MRU VARIO). The generated NO_*x*_ was continuously fed into the electroreduction cells (single or tandem) for 1 h, absorbed by the alkaline electrolyte, and catalyzed into NH_3_.

### Catalyst Synthesis

VG arrays were first prepared using an RF PECVD method powered by a high-frequency generator (Kmate, HERO-500 W, 13.56 MHz). A piece of CC was used as the substrate and thoroughly rinsed before use. First, the CC was placed in a quartz tube with a diameter of 48 mm and a length of 1000 mm, and the entire process was carried out under low-pressure conditions (∼0.5 Torr), which is vital for the growth of graphene. Subsequently, a mixed gas of methane (CH_4_), hydrogen (H_2_), and argon (Ar) was then introduced at a total flow rate of 35 mL min^–1^ (CH_4_: 20 mL min^–1^; H_2_: 5 mL min^–1^; Ar: 10 mL min^–1^). The RF plasma was generated at 500 W for 10 min, resulting in the decoration on the CC substrate.

MoS_2_ nanosheets were fabricated using a conventional hydrothermal method. The VGs obtained were immersed in a homogeneous solution containing 4.8 mmol of Na_2_MoO_4_·2H_2_O (sodium molybdate dihydrate), 20 mmol of CH_3_CSNH_2_ (thioacetamide), and 80 mL of deionized water after vigorous stirring for 30 min. Then the mixture was transferred to a Teflon-lined autoclave, and the temperature was maintained at 200 °C for 12 h. The obtained sample was then annealed at 300 °C to obtain MoS_2_/VG arrays. The MoS_2_/VGs sample was further treated with RF plasma under an NH_3_ atmosphere at a flow rate of 40 mL min^–1^ for 20 min to obtain N-MoS_2_/VGs.

### Catalyst Characterizations

Field-emission SEM images were recorded using a Hitachi SU8010. TEM images, EDX elemental mappings, and a SAED pattern were collected using a JEOL 2100F microscope. HAADF images were collected using a JEM-ARM200F microscope. XRD patterns were obtained using Cu Kα radiation on a Rigaku D/Max-2550 diffractometer. Raman spectra were collected using a Renishaw-in Via Raman microscope with 514 nm laser excitation. XPS was obtained using an ESCALAB_250Xi X-ray photoelectron spectrometer. Specific surface area distributions were determined using a JW-BK112 analyzer. EPR spectra were taken using a JES FA-200 continuous-wave spectrometer with a sweeping magnetic field and an X band (9.2 GHz) at room temperature. Details of these methods are available in SI.

## Supporting Information Available

Additional experimental details and methods; energy consumption calculations; phase transformation and kinetic enhancement enabled by plasma engraving; calibration curves of NH_4_^+^ and N_2_H_4_ and ^1^H NMR spectra, chronoamperometry curves, UV–vis absorption spectra, and NRR performance for each reaction; morphology of the catalyst before and after stability tests; ammonia quantification by ^15^N and ^14^N isotope labeling; DFT calculation details; energy consumption comparison of plasma NO_*x*_ generation and eNO_2_-RR; summary of reported ammonia syntheses in conventional eNRR and plasma tandem-electrocatalysis systems; and summary of reported solar-powered plasma setups and solar-driven electrocatalysis and tables of performance comparison (PDF)
