# Supplementary material for: Enhanced NH3 Synthesis from Air in a Plasma Tandem-Electrocatalysis System Using Plasma-Engraved N-Doped Defective MoS2
Source: JACS Au. 2023 Apr 26;3(5):1328–36. doi: 10.1021/jacsau.3c00087 (PMC10207100; doi:10.1021/jacsau.3c00087)
Supplement: Supplementary file 1 — au3c00087_si_001.pdf [file au3c00087_si_001.pdf]

Supporting Information

Enhanced NH_3 synthesis from air in a plasma tandem-electrocatalysis system using plasma-engraved N-doped defective MoS_2

Jiageng Zheng [a‡], Hao Zhang [a*,‡], Jiabao Lv[a], Meng Zhang[b], Jieying Wan[a], Nick Gerrits[c], Angjian Wu[a], Bingru Lan[a], Weitao Wang[d], Shuangyin Wang[e*], Xin Tu[d*], Annemie Bogaerts[c*], and Xiaodong Li[a]

[a] State Key Laboratory of Clean Energy Utilization, College of Energy and Engineering, and Academy of Ecological Civilization, Zhejiang University, Hangzhou 310027, China

[b] College of Optical Science and Engineering, Zhejiang University, Hangzhou 310027, China

[c] Research group PLASMANT, Department of Chemistry, University of Antwerp, Universiteitsplein 1, Wilrijk BE-2610, Belgium

[d] Department of Electrical Engineering and Electronics, University of Liverpool, Liverpool, L69 3GJ, UK

[e] State Key Laboratory of Chem/Bio-Sensing and Chemometrics, College of Chemistry and Chemical Engineering, Hunan University, Changsha, 410082, China

‡These authors contributed equally.

*Corresponding authors:

zhang_hao@zju.edu.cn (Hao Zhang)

shuangyinwang@hnu.edu.cn (Shuangyin Wang)

xin.tu@liv.ac.uk (Xin Tu)

annemie.bogaerts@uantwerpen.be (Annemie Bogaerts)

Table of contents

S-I: Additional experimental details and methods.

S-I-1: Experimental details.

S-I-2: Electrochemical evaluation.

S-I-3: Quantification of NH_4^+, N_2H_4 and ^1H NMR.

S-II: Energy consumption (EC) calculations.

S-II-1: EC calculation for plasma NO_x generation.

S-II-2: EC calculation for eNO_2^-RR.

S-II-3: Calculation of the Faradic efficiency (FE) and ammonia production rate.

S-III: Phase transformation and kinetic enhancement enabled by plasma engraving.

S-III-1: HRTEM images of phase transformation from 2H to 1T.

S-III-2: Element contents of N-MoS_2/VGs and MoS_2/VGs from EDX.

S-III-3: Comparison of the electrochemical properties of various catalysts.

S-IV: The calibration curves of NH_4^+, N_2H_4 and ^1H NMR, chronoamperometry curves, UV-Vis absorption spectra, and NRR performance for each reaction.

S-IV-1: The calibration curves of NH_4^+, N_2H_4 and ^1H NMR.

S-IV-2: Chronoamperometry curves, UV-Vis absorption spectra, and NRR performance for each reaction.

S-IV-3: UV-vis absorption spectra of N_2H_4 after electrolysis.

S-V: Morphology of the catalyst before and after stability tests.

S-VI: Ammonia quantification by ^{15}N and ^{14}N isotope-labeling.

S-VII: DFT Calculation details.

S-VII-1: Calculation methods.

S-VII-2: MoS_2 (100) slab models.

S-VII-3: The differential charge density of adsorbed NO_2^- of 2H-MoS_2 and 1T-MoS_2 with different iso-surface value.

S-VII-4: PDOS plots of 2H-MoS_2 and 1T-MoS_2.

S-VII-5: Selected adsorption sites for NO_2^- and H atoms on MoS_2 (100).

S-VII-6: Free energy calculation of N-MoS_2 for N_2 adsorption and VGs for NO_2^- adsorption.

S-VII-7: The Gibbs free energy changes of various eNO_2^-RR pathways.

S-VII-8: HER and eNO_2^-RR Gibbs free energy diagrams of 1T-MoS_2 and 2H-MoS_2

S-VIII: Energy consumption comparison of plasma NO_x generation and eNO_2-RR.

S-IX: Summary of reported ammonia synthesis in conventional eNRR and plasma tandem-electrocatalysis systems.

S-X: Summary of reported solar powered plasma setups, and solar-driven electrocatalysis.

S-I: Additional experimental details and methods.

S-I-1: Experimental details.

The details of two comparative experiments are as follows:

1. The plasma gas was fed into the electrolyte for 1 h during the plasma alone process. The electrolyte was then collected for further analysis. The entire procedure was carried out without the use of electrocatalysis.
2. In the electrocatalysis alone process, the gas (air) was fed into the electrolyte with the plasma turned off. However, the electrochemical workstation was turned on for 1 h to carry out the electrocatalysis process. The electrolyte was then collected for further analysis. The entire procedure was carried out without the use of plasma.
3. In our system, the typical power in the plasma is around 176 W. Two electrolytic cells were used as the cascaded electrocatalytic device aiming for elevated production rate. More specifically, the first cell acts as the main nitrogen fixation reaction chamber and an extra cell served as both ammonia trapper and nitrogen oxides absorber.
4. The split cells (H type cells) are two cells (cathode cell and anode cell) separated by the proton exchange membrane (PEM), which allows the eNO_2^-RR at cathode cell and OER at anode cell to proceed individually. Also, the products (NH_3 and O_2) of the counter reaction (eNO_2^-RR and OER) can be separated in the H type cells.
5. The remaining Faradaic efficiency is most likely due to the competing HER reaction, since the entire process involved only eNO_2^-RR and HER, and no liquid byproducts (e.g., N_2H_4) were detected (see Section S-IV-3 in SI). Nevertheless, the H_2 produced was not measured due to its very low concentration caused by a high flow rate (9 SLM). But we will measure H_2 concentration in our future work by using GC, as suggested by the reviewer.

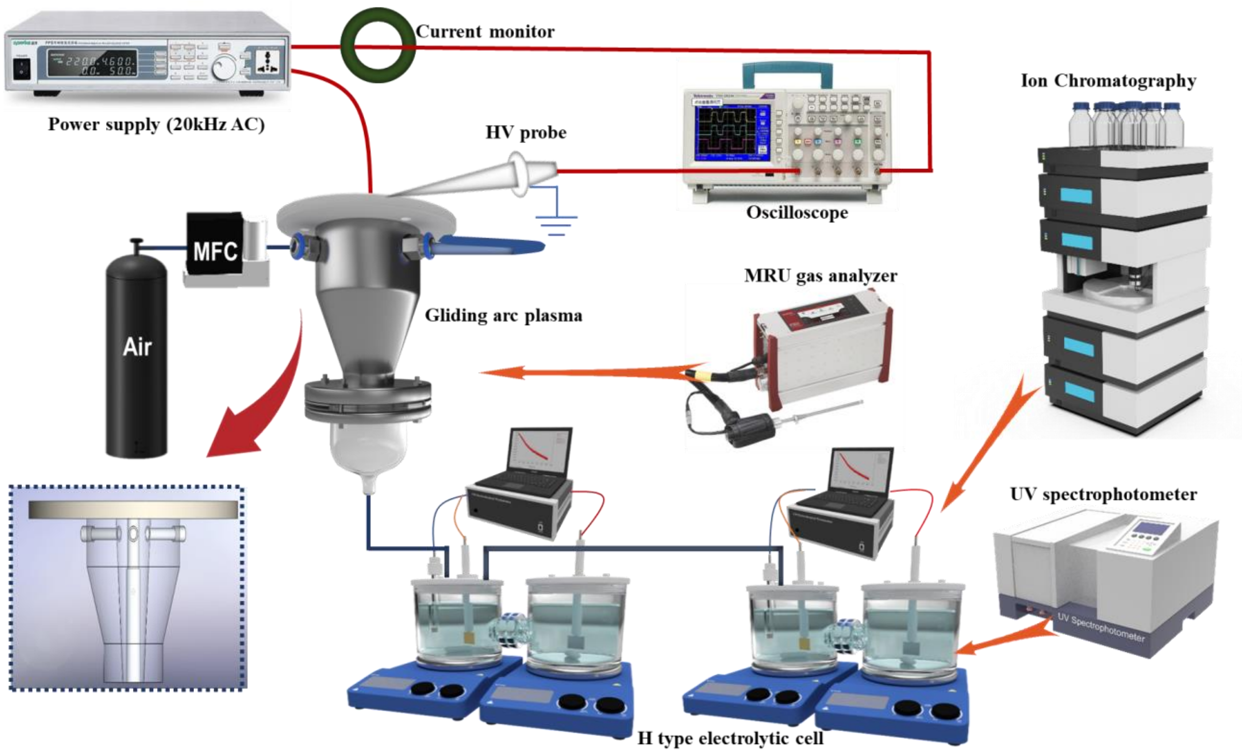

Figure S1. Schematic of the plasma tandem-electroreduction system for sustainable ammonia production.

S-I-2: Electrochemical evaluation.

All the electrochemical tests were conducted using two H-type cells (GAOSSUNION) connected in series with well-sealed rubber rings and stainless-steel tubes and the chambers of each cell were separated by a proton exchange

membrane (DuPont, Nafion 117). All data were collected with a typical three-electrode system connected to a CHI-660E electrochemical workstation (CHI Instrument, Inc.). The as-prepared electrocatalysts, Ag/AgCl, and platinum foil were used as the working, reference, and counter electrodes, respectively. The potentials were converted into the RHE according to the equation: $E_{RHE} = E_{Ag/AgCl} + 0.207\ V + 0.0591 \times pH$. A solution with 0.1M KOH was used as the electrolyte and NO_x absorbent. The two H-cell had volumes of 200 ml and 80 ml, respectively, and were purged with pure argon for 20 minutes before testing. The cyclic voltammetry (CV) curves were performed at a scan rate of 5 mV s^{-1} and electrochemical impedance spectroscopy (EIS) was carried out in a frequency range of 1 MHz to 10 mHz with an AC amplitude of 10 mV. Catalysts with a geometric area of 1 × 1 cm^2 were used for potentiostatic tests at various potentials (- 0.13 ~ -0.53 V vs RHE) for 1 h at a stirring rate of 280 rpm. The electrochemically active surface area (ECSA) was calculated from the following equations: ECSA = C_{dl}/C_s, where C_s is the specific capacitance of an atomically smooth planar surface and is considered to be 40 μF cm^{-2} for a 0.1M KOH medium.

S-I-3: Quantification of NH_4^+, N_2H_4 and ^1H NMR.

Ammonia quantification by typical indophenol blue method.

The quantification of ammonia production was conducted by the typical indophenol blue method. 0.5 mL of the reacted solution was extracted from the cathode chamber, and then diluted to 4 mL for the following detection. Afterwards, 0.32 mL of 1 wt% sodium nitroferricyanide ($Na_2[Fe(CN)_5NO]\cdot 2H_2O$) aqueous solution was added into diluted samples, followed by addition of 2.4 mL of 0.32 M NaOH solution containing 10.4 M sodium salicylate ($C_6H_4(OH)COONa$), and 0.8 mL of 0.3 M NaClO and 0.75 M NaOH mixture. The indophenol blue absorbance was measured using an Ultraviolet-visible (UV–vis) spectrophotometer (MAPADA, UV-1800) after 2 hours of incubation. For reference, the calibration was built with standard ammonia chloride solution (0.5 - 5 μg ml^{-1}).

Hydrazine quantification by Watt and Chrisp method.

The ammonia production was quantified using a Watt and Chrisp method. The coloring agent was prepared by mixing 5.99 g of para-(dimethylamino)benzaldehyde (p-$C_9H_{11}NO$) with 30 mL of hydrochloric acid (HCl) and 300 mL of ethanol (C_2H_5OH). Then, 9 mL of 1.0 M HCl was added to 1 mL reacted solution, followed by the addition of 5 mL of coloring agent. The absorbance was measured using the aforementioned UV–vis spectrophotometer after 30 min of incubation. For reference, the calibration was built with standard hydrazine solution (1 - 5 μg ml^{-1}).

Nitrate and nitrite (NO_3^- and NO_2^-) quantification.

First, the reacted solution was extracted from the cathode chamber and diluted to detectable levels. 1 ml of the sample was measured by ion chromatography (IC, ICS-3000). A series of standard potassium nitrate and potassium nitrite solutions are used to obtain the concentration–intensity curves.

Nuclear magnetic resonance (NMR) analysis.

Ammonia and ^{15}N isotope-labeling were also quantitatively determined by ^1H nuclear magnetic resonance (NMR, 600 MHz) using maleic acid ($C_4H_4O_4$) as the internal standard. Firstly, the calibration curve was established as follows: 40 ml 0.1 M KOH (with a standard concentration of 1000, 500, 250, 125 ppm ammonia chloride); Secondary, the pH of the above solution was adjusted by adding dropwise to 2 ~ 3; Afterwards, the maleic acid (with the concentration kept at 100 ppm) was added into the mix solution and the acidulated solution was tested by a 600 MHz liquid Agilent DD2 NMR spectrometer at room temperature; Finally, the calibration curve was achieved using the peak area ratio between NH_4^+ and $C_4H_4O_4$ because the NH_4^+ concentration and the area ratio were positively correlated.

As for the ammonia and ^{15}N detection, the process was the same as explained above, except that the standard concentration of 1000, 500, 250, 125 ppm ammonia chloride was replaced by the reacted solution.

S-II: Energy consumption (EC) calculation.

S-II-1:EC calculation for plasma NO_x generation.

The electrical signals of the discharge were recorded by a digital oscilloscope (Tektronix DPO4034B). The average discharge power (P_1) was calculated with the measured voltage (U_1) and current (I_1), as shown in equation 1.

$$P_1 = U_1 \times I_1 \qquad (S1)$$

$$EC\ [MJ\ mol^{-1}] = P_1\ [W] / (NO_x\ \text{production rate}\ [mol\ s^{-1}] \times 10^6) \qquad (S2)$$

S-II-1:EC calculation for eNO_2^-RR.

The electric power converting NO_x intermediaries to ammonia is calculated by Eq. (S3), where P_2 is power (W), U_2 is applied potential (V) and I_2 is current (A). In the electrolyser, a certain voltage was applied, and the output current was recorded. We took the average current multiplied by the applied voltage to calculate the power.

$$P_1 = U_1 \times I_1 \qquad (S3)$$

$$EC\ [MJ\ mol^{-1}] = P_2\ [W] / (NH_3\ \text{production rate}\ [mol\ s^{-1}] \times 10^6) \qquad (S4)$$

S-II-3: Calculation of the Faradic efficiency (FE) and ammonia production rate.

The FE of electrocatalytic NO_2^-, NO_3^-, and NO to NH_3 conversion were calculated as follows:

Main: $$FE_{NH3} = (6 \times F \times C_{NH3} \times V) / (17 \times Q) \qquad (S5)$$

Minor: $$FE_{NH3} = (8 \times F \times C_{NH3} \times V) / (17 \times Q) \qquad (S6)$$

$$FE_{NH3} = (5 \times F \times C_{NH3} \times V) / (17 \times Q) \qquad (S7)$$

The ammonia production rate was calculated as follows:

$$v_{NH3} = (C_{NH3} \times V) / (t \times S_{cat.}) \qquad (S8)$$

where F is the Faraday constant (96,485 C mol^{-1}), C_{NH3} is the measured NH_3 concentration, V is the volume of the cathodic electrolyte, Q is the total charge passing the electrode, t is the reduction time, and $S_{cat.}$ is the total area of catalysts. Note: In this study, the FE was calculated based on function (S5).

S-III: Phase transformation and kinetic enhancement enabled by plasma engraving.

S-III-1: HRTEM images of phase transformation from 2H to 1T.

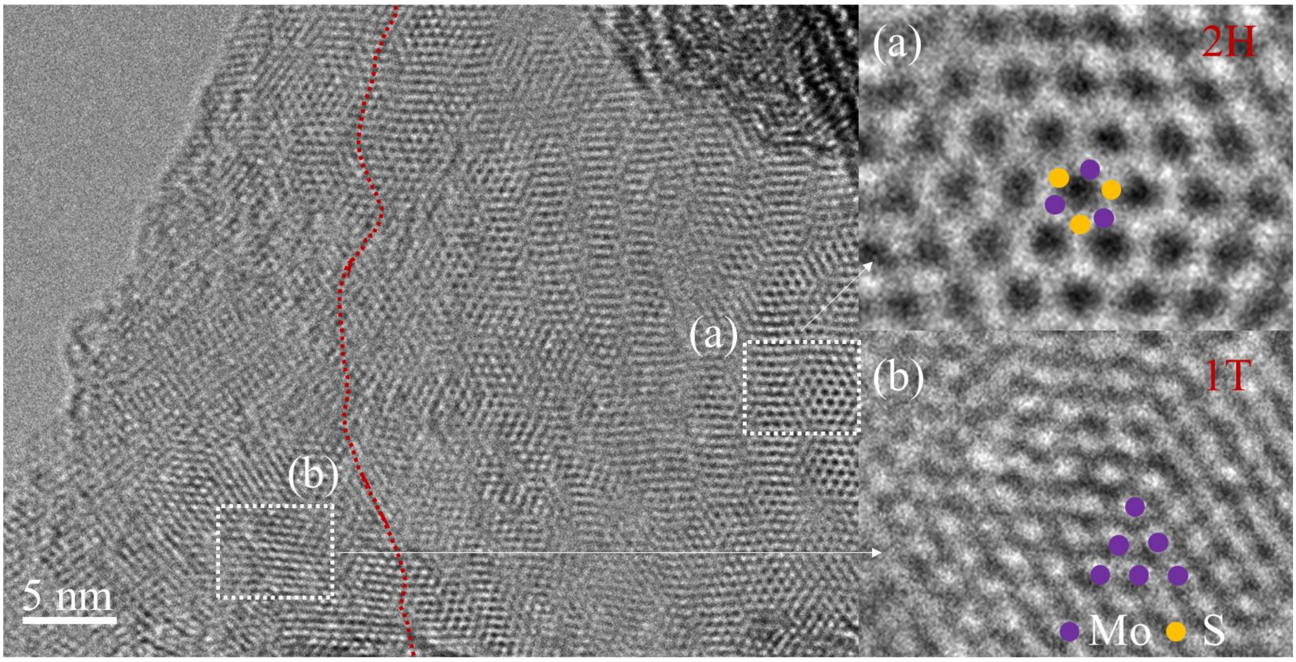

Figure S2. HRTEM images of MoS_2/VGs: the regions enclosed by the white rectangle are enlarged in (a) 2H-MoS_2, (b) 1T- MoS_2 and the red line divides the boundary of 1T and 2H phase.

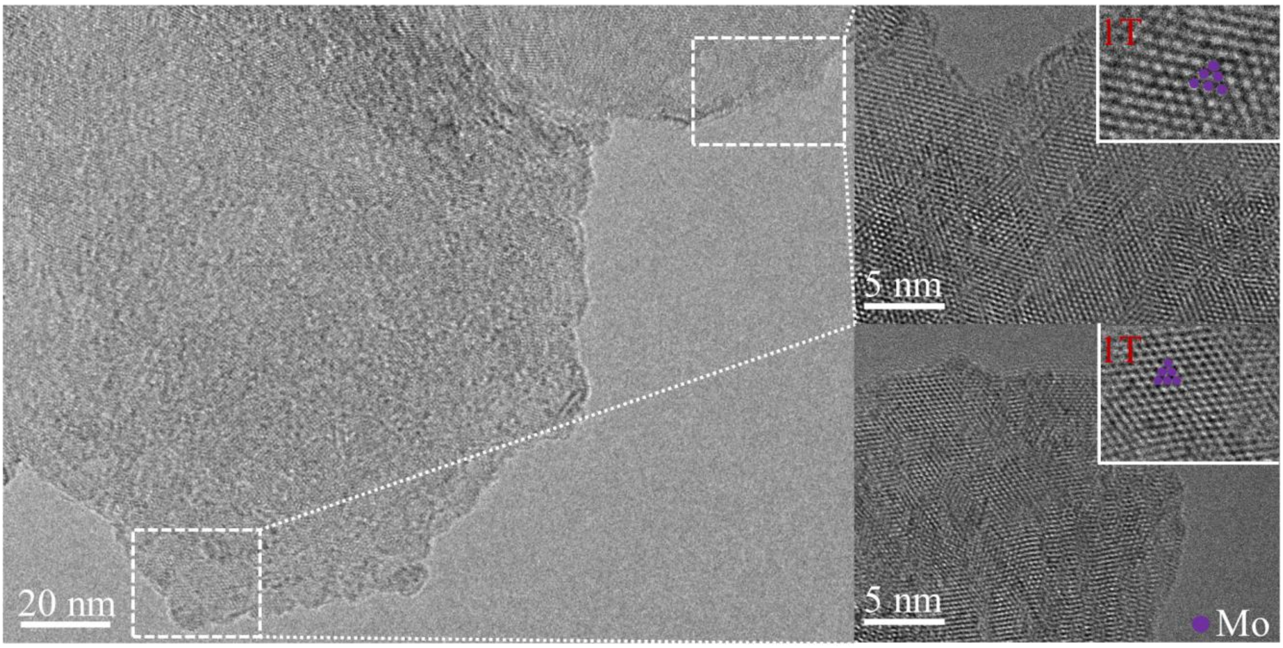

Figure S3. HRTEM images of N-MoS_2/VGs: the various regions selected with the white rectangle are evidenced to be a high degree of 1T-MoS_2. The phase transformation of MoS_2 from 2H to 1T has delivered by HRTEM images as shown in Fig. S2-S3: the degree of 1T phase MoS_2 is obviously increased enabled by the plasma engraving.

S-III-2: Element contents of N-MoS_2/VGs and MoS_2/VGs from EDX.

Table S1. Element contents of N-MoS_2/VGs and MoS_2/VGs from EDX.

Sample	Mo (wt %)	S (wt %)	Mo/S
N-MoS_2	63.8	33.2	1.37
MoS_2	56.1	37.2	1.74

S-III-3: Comparison of the electrochemical properties of various catalysts.

Table S2. The electrochemical properties of CC, VGs, MoS_2/VGs, and N-MoS_2/VGs.

Catalyst	S_{BET} (m^2 g^{-1})	C_{dl} (mF cm^{-2})	ECSA (cm^2)	R_{ct} (Ω)
CC	/	1.1	18.8	~18
VGs	/	1.2	20.3	~15
MoS_2	49.9	8.0	133.5	~2
N-MoS_2	58.5	9.7	162.2	<1

The results revealed that the N-MoS_2/VGs possess the largest specific surface area (S_{BET}), the largest value of double-layer capacitance (C_{dl}), the largest electrochemically active surface area (ECSA) and the smallest electrochemical impedance.

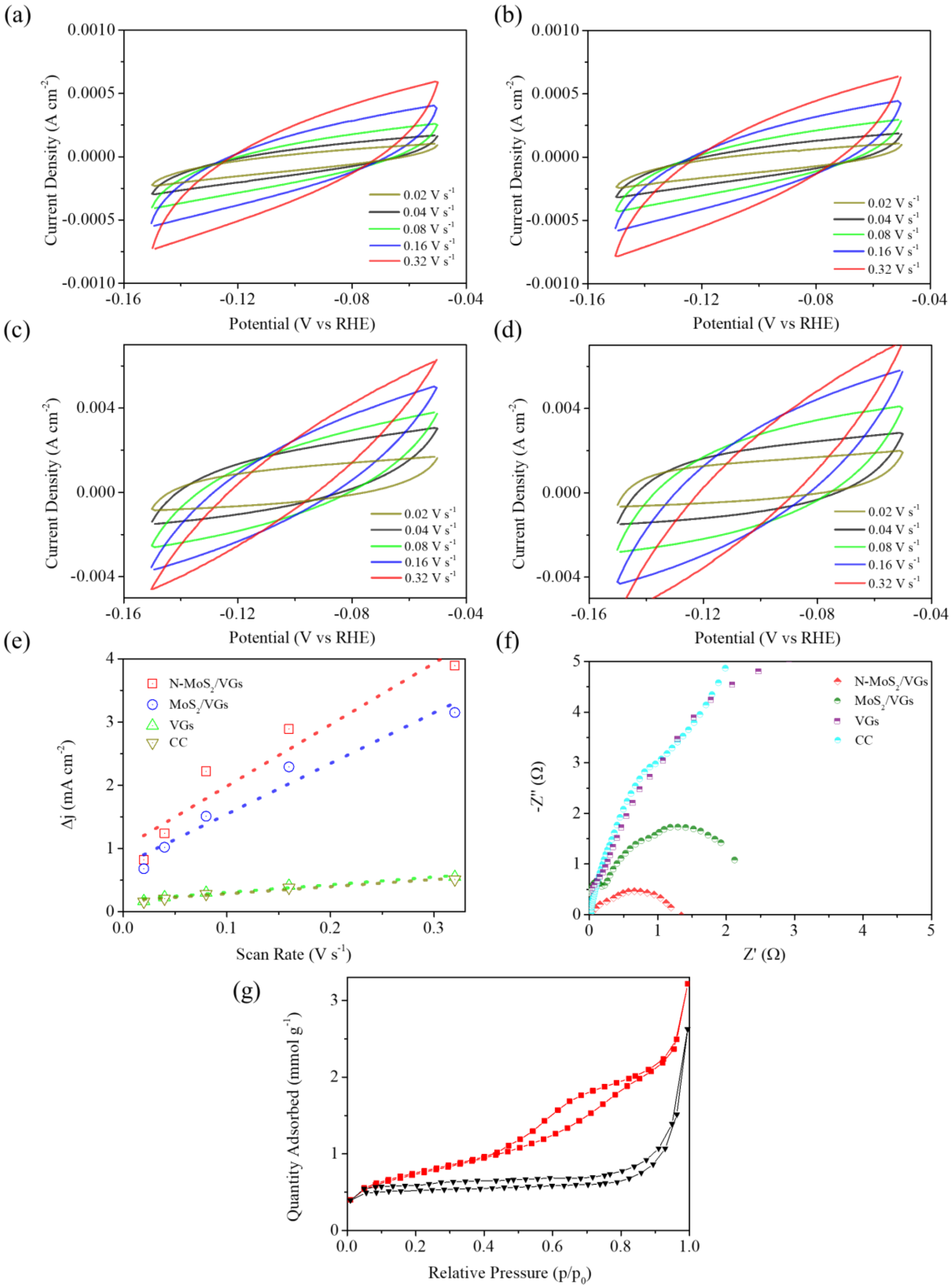

Figure S4. Cyclic voltammetry (CV) curves of (a) CC, (b) VGs and (c) MoS_2/VGs and (d) N-MoS_2/VGs in double layer region at various scan rates ranging from 0.02 to 0.32 V s^{-1}, (e) the ratio of current density with various scan rates, (f) Nyquist plots of CC, VGs, MoS_2/VGs, and N-MoS_2/VGs. (g) BET measurements: nitrogen adsorption-desorption isotherm curves of N-MoS_2/VGs (red curve) and MoS_2/VGs (black curve). This figure shows that the N-MoS2/VGs has clearly better kinetic activity.

S-IV: The calibration curves of NH_4^+, N_2H_4 and ^1H NMR., chronoamperometry curves, UV-Vis absorption spectra, and NRR performance for each reaction.

S-IV-1: The calibration curves of NH_4^+, N_2H_4 and ^1H NMR.

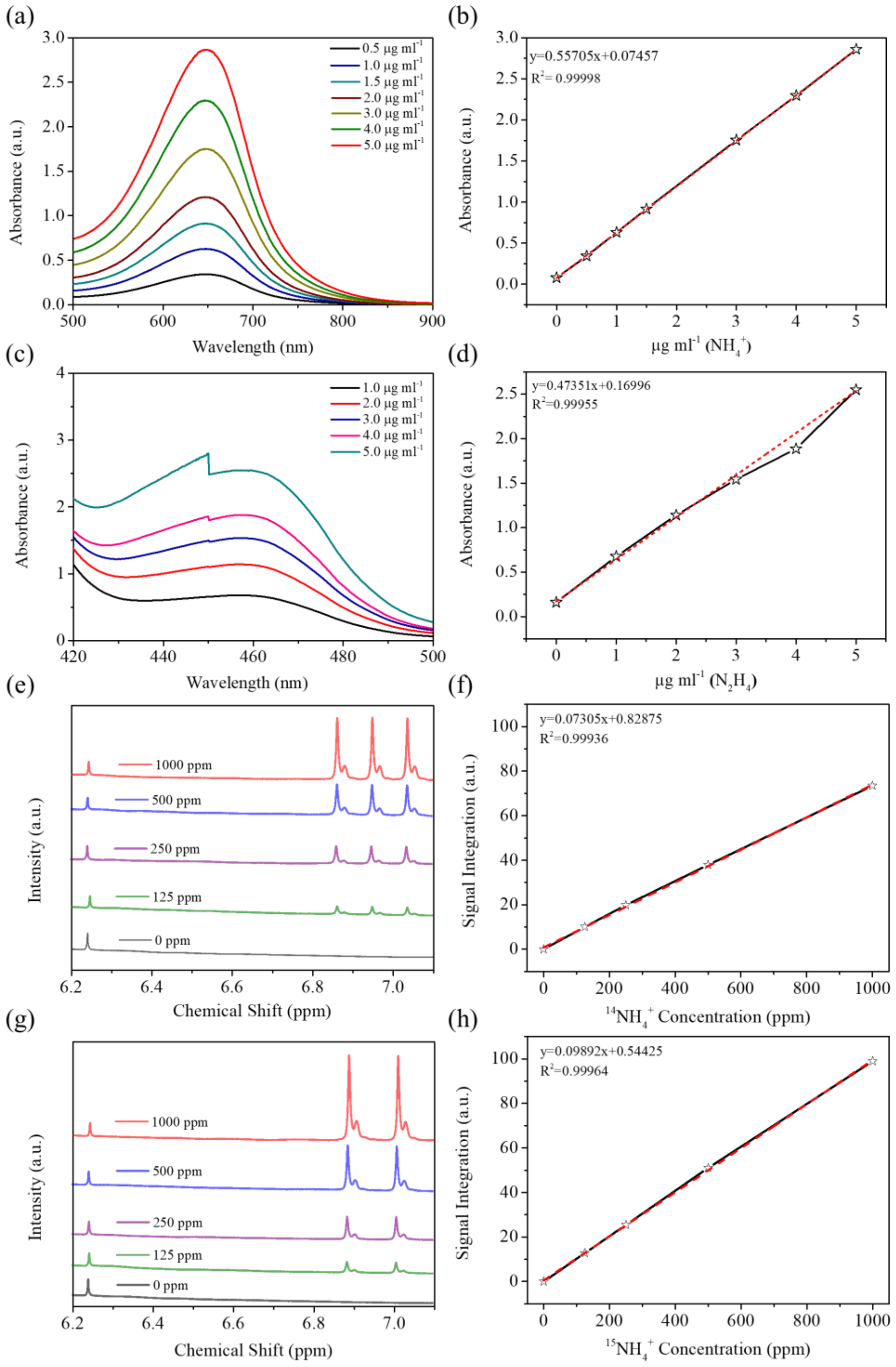

Figure S5. (a) UV-Vis absorption spectra of indophenol blue indicator with NH_4^+ after incubation for 2 h at room temperature. (b) Calibration curve used for estimation of NH_4^+ concentrations. (c) UV-Vis absorption spectra of various N_2H_4 concentrations after incubation for 30 min at room temperature. (d) Calibration curve used for quantification of N_2H_4 concentrations. (e) NMR spectra of the standard solutions with different concentrations of $(^{14}NH_4)_2Cl$. (f) Calibration curves for the $^{14}NH_4^+$. (g) NMR spectra of the standard solutions with different concentrations of $(^{15}NH_4)_2Cl$. (h) Calibration curves for the $^{15}NH_4^+$.

S-IV-2: Chronoamperometry curves, UV-Vis absorption spectra, and NRR performance for each reaction.

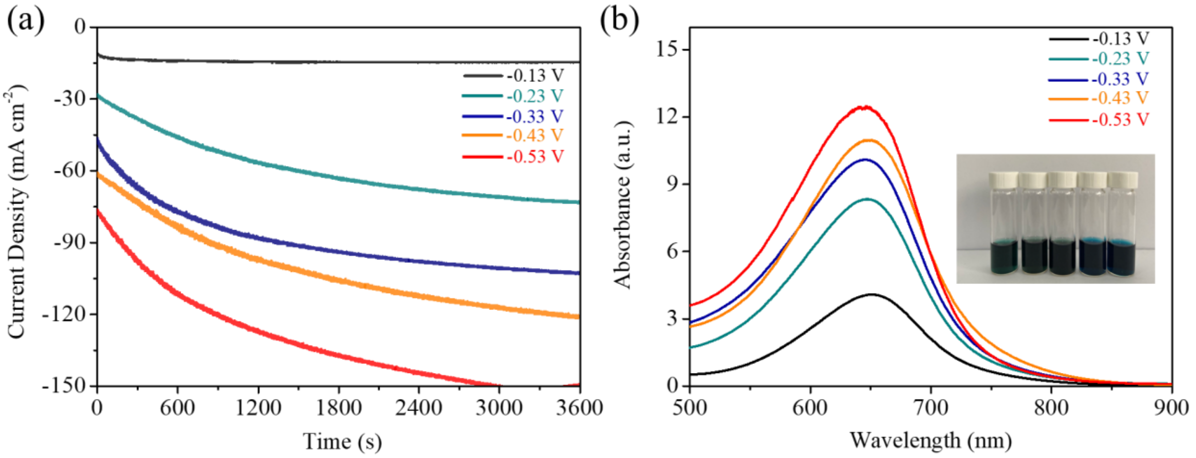

Figure S6. (a) Chronoamperometry curves for N-MoS_2/VGs at various potentials in plasma single-electrocatalysis mode. (b) UV-Vis absorption spectra of the electrolyte stained with indophenol blue indicator after electrolysis at selected potentials of N-MoS_2/VGs in plasma single-electrocatalysis mode.

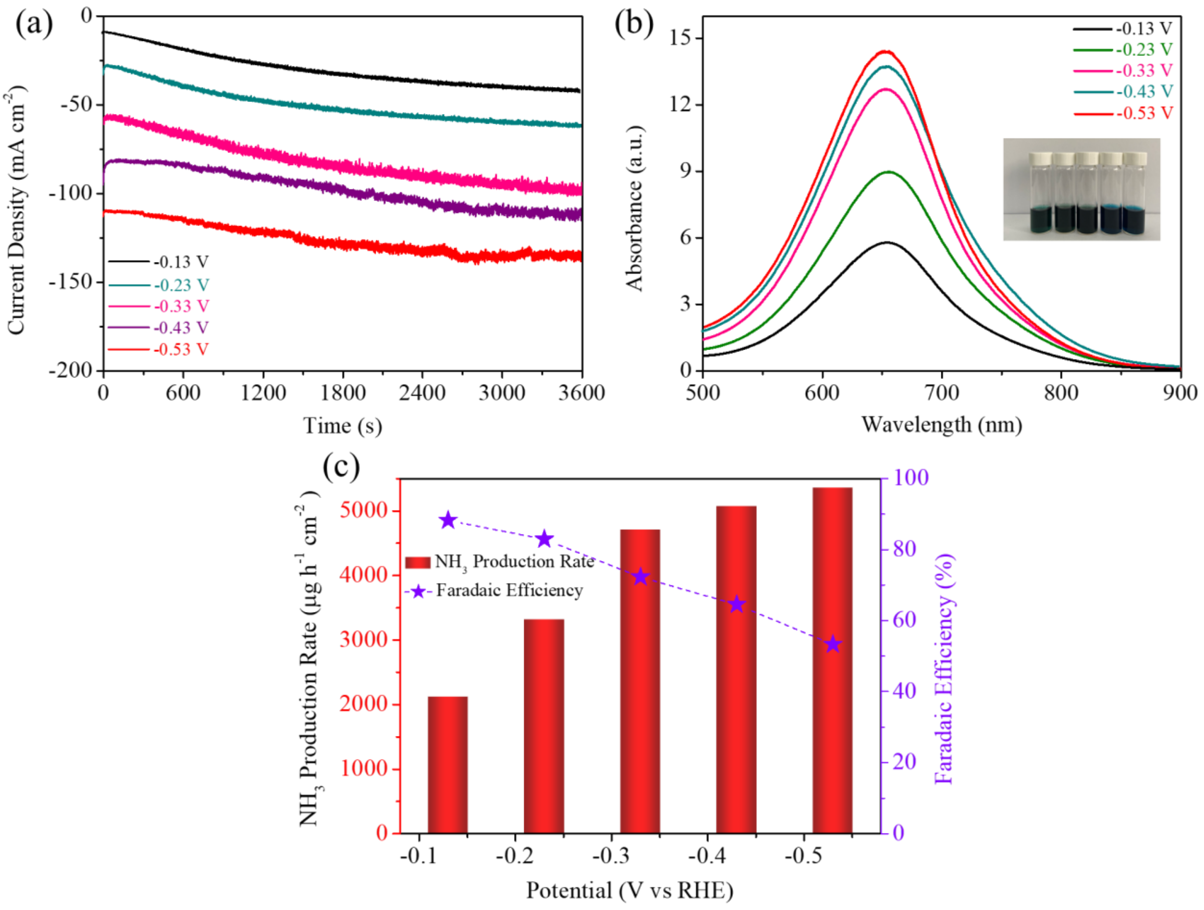

Figure S7. (a) Chronoamperometry curves for N-MoS_2/VGs at various potentials of first cell in plasma tandem-electrocatalysis mode. (b) UV-Vis absorption spectra of the electrolyte stained with indophenol blue indicator after electrolysis at selected potentials for N-MoS_2/VGs of first cell in plasma tandem-electrocatalysis mode. (c) NH_3 production rates and corresponding FEs for N-MoS_2/VGs of the first cell in plasma tandem-electroreduction mode.

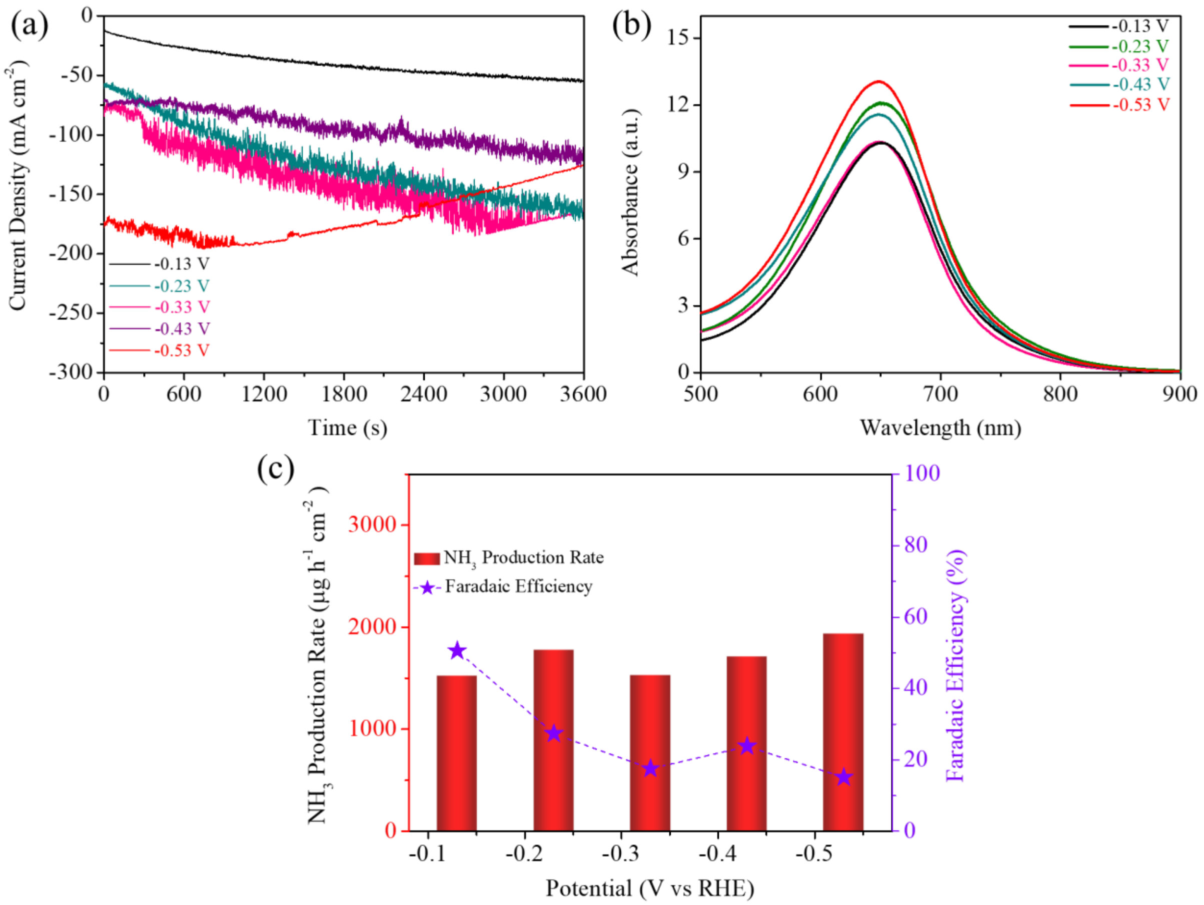

Figure S8. (a) Chronoamperometry curves for N-MoS_2/VGs at various potentials of second cell in plasma tandem-electrocatalysis mode. (b) UV-Vis absorption spectra of the electrolyte stained with indophenol blue indicator after electrolysis at selected potentials for N-MoS_2/VGs of second cell in plasma tandem-electrocatalysis mode. (c) NH_3 production rates and corresponding FEs for N-MoS_2/VGs of second cell in plasma tandem-electrocatalysis mode.

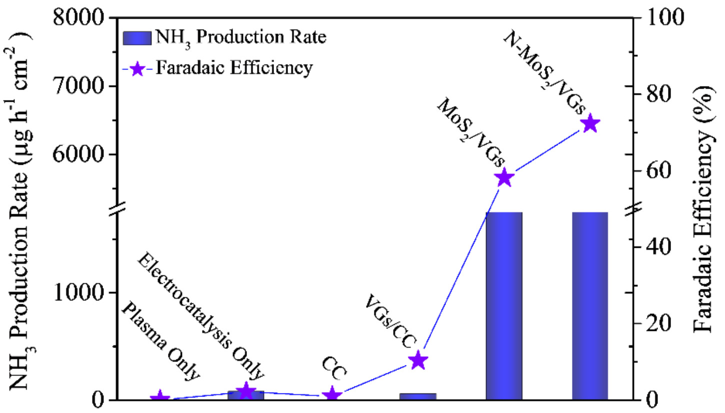

Figure S9. NH_3 production rates and corresponding FEs for various electrocatalysts at the potential of -0.33 V vs RHE and electrocatalysis or plasma performed alone in first cell of plasma tandem-electrocatalysis mode.

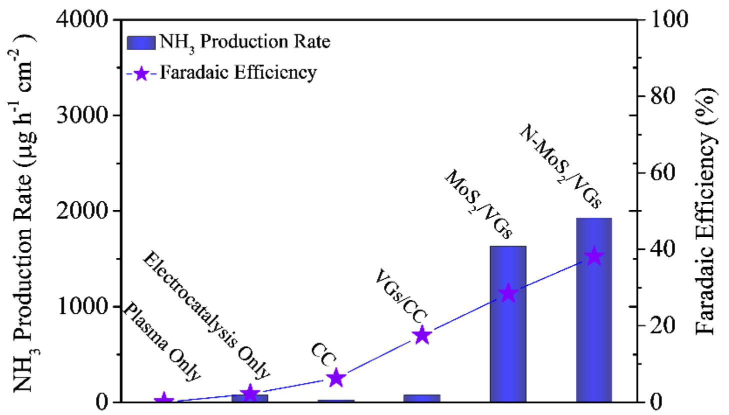

Figure S10. NH_3 production rates and corresponding FEs for various electrocatalysts at the potential of -0.33 V vs RHE and electrocatalysis or plasma performed alone in second cell of plasma tandem-electrocatalysis mode.

S-IV-3: UV-vis absorption spectra of N_2H_4 after electrolysis.

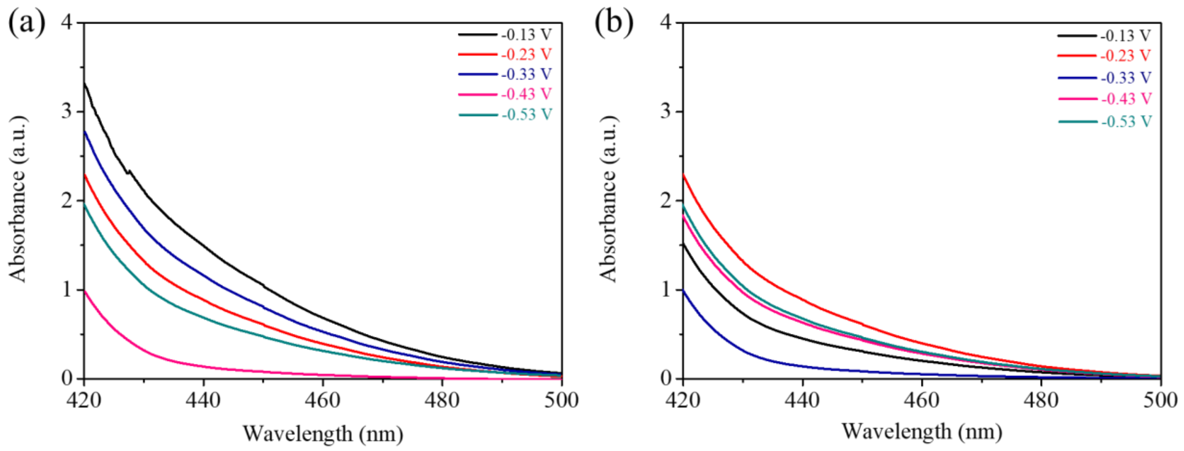

Figure S11. UV-vis absorption spectra of N_2H_4 in 0.1 M KOH electrolyte after electrolysis on N-MoS_2/VGs electrode stained with N_2H_4 color indicator after 0.5 h at various potentials for (a) first cell and (b) second cell of plasma tandem-electrocatalysis mode. No hydrazine was detected during the reaction, indicating that NO_x^- was selectively reduced to NH_3.

S-V: Morphology of the catalyst before and after stability tests.

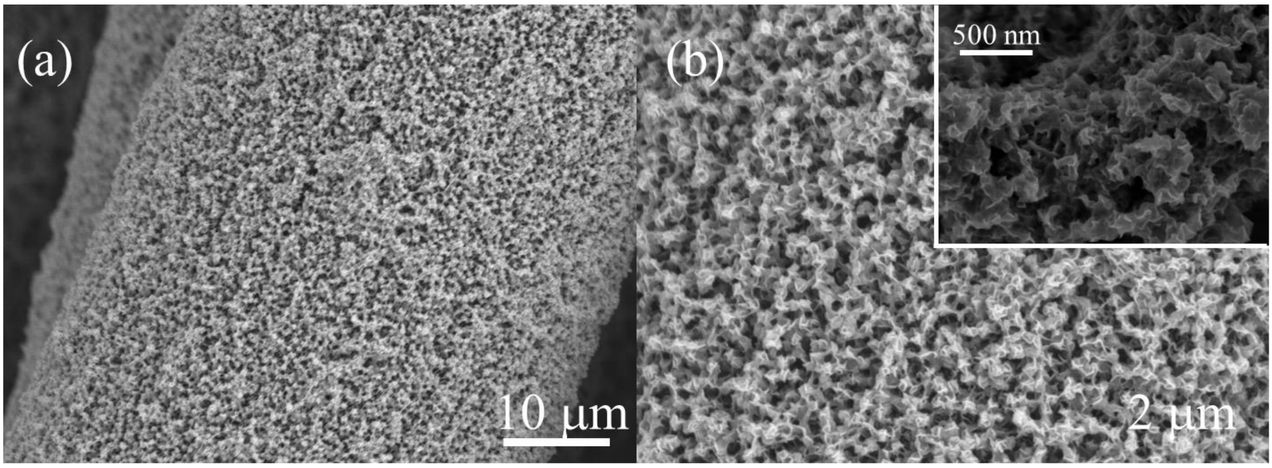

Figure S12. SEM images of N-MoS_2 supported by carbon cloth with different magnifications before stability tests: (a) 10 μm, (b) 2 μm, and (insert) 500 nm. The SEM images reveal that VGs are uniformly covered by MoS_2 arrays. The crinkly VGs provides larger area to support the deposition and self-growth of MoS_2 during the HT process.

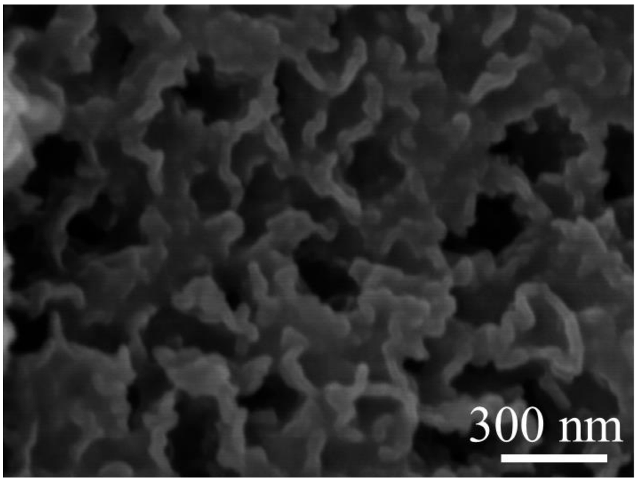

Figure S13. SEM images of N-MoS_2 after 24 h stability tests.

S-VI: Ammonia quantification by ^{15}N and ^{14}N isotope-labeling.

^{15}N isotope-labeling experiment.

To demonstrate that the produced ammonia originates from the source of N_2 gas rather than potential contamination, $^{15}N_2$ isotopic experiments were carried out, with the ammonia detected by ^{1}H NMR to distinguish between $^{15}NH_4^+$ and $^{14}NH_4^+$. The NMR test method was listed in section S-IV-4: "Nuclear magnetic resonance (NMR) analysis". The $^{15}NH_4^+$ and $^{14}NH_4^+$ production rates are almost the same, excluding the influence of the surroundings or the plasma, and demonstrating that the produced ammonia effectively originates from the source of N_2 gas rather than potential contamination.

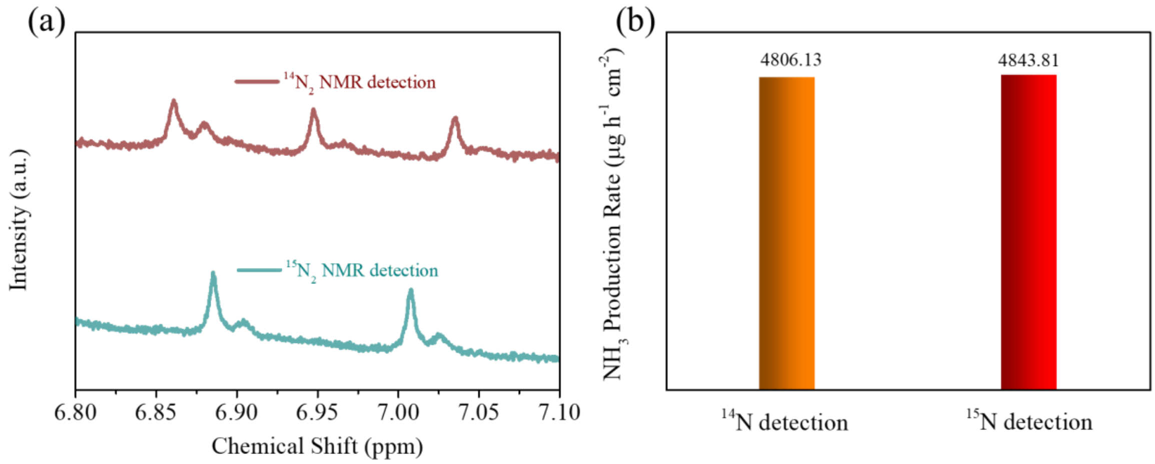

Figure S14. (a) NMR spectra of the $^{14}NH_4^+$ and $^{15}NH_4^+$ detection and (b) corresponding NH_3 production rates comparison.

S-VII: DFT Calculation details.

S-VII-1: Calculation methods.

All the first-principles calculations were performed by the "Vienna ab initio simulation package" (VASP)[2] with the theoretical basis of density functional theory (DFT). Generalized gradient approximation (GGA) with Perdew, Burke, and Ernzerhof[3] (PBE) was adopted to treat the exchange and correlation effects. The nuclei-electron was described by the projector augmented wave (PAW) pseudopotentials. The van der Waals interactions were included using the DFT-D3 method. We utilized the Gaussian smearing method and set the value of SIGMA as 0.05. The bandgap error of MoS_2 in the DOS calculation was corrected by DFT + U (U=2.0eV). The cut-off energy for the plane-wave basis was set at 400 eV. 10^{-6} eV and -0.05 eV $Å^{-1}$ were set as the convergence criteria for total energy and the Hellman-Feynman force. A 3-layer (3×2) MoS_2 (100) bulk was built to simulate the supercell model and optimized the lattice parameters for convergence. The bottom layer of the z-axis was fixed to improve computational efficiency. The surface atoms of MoS_2 were exposed with a sufficient vacuum gap of 20 Å in the z-direction to simulate the reaction on the catalyst surface, as shown in Figure S15. A 3 × 3 × 1 k-point grid was set in the first Brillouin zone. The adsorption energy (E_{ads}) was defined as:

$$E_{ads} = E_{slab} + E_{mol} - E_{total} \quad (S9)$$

Where E_{slab}, E_{mol}, and E_{total} represent the energy of the isolated slab module, the energy of isolated species, and the energy of the species-adsorbed system.

The synthesis of ammonia was described by the fundamental reactions:

$$* + NO_2^- \rightarrow *NO_2^- \quad (S10)$$

$$* + NO_2^- + (H^+ + e^-) \rightarrow *NO_2H \quad (S11)$$

$$*NO_2H + (H^+ + e^-) \rightarrow *NO + H_2O \quad (S12)$$

$$*NO + H_2O + (H^+ + e^-) \rightarrow *NHO + H_2O \quad (S13)$$

$$*NHO + H_2O + (H^+ + e^-) \rightarrow *NHOH + H_2O \quad (S14)$$

$$*NHOH + H_2O + (H^+ + e^-) \rightarrow *NH + 2H_2O \quad (S15)$$

$$*NH + 2H_2O + (H^+ + e^-) \rightarrow *NH_2 + 2H_2O \quad (S16)$$

$$*NH_2 + 2H_2O + (H^+ + e^-) \rightarrow *NH_3 + 2H_2O \quad (S17)$$

$$*NH_3 + 2H_2O \rightarrow * + NH_3 + 2H_2O \quad (S18)$$

And the total reaction path can be set as:

$$* + NO_2^- + 7(H^+ + e^-) \rightarrow * + NH_3 + 2H_2O \quad (S19)$$

The Gibbs free energy change (ΔG) can be obtained as follows:

$$\Delta G = \Delta E + \Delta ZPE - T\Delta S \quad (S20)$$

Where ΔE represents the electronic energy difference. ΔZPE and TΔS represent the change in zero-point energies and the product of temperature (298.15K) and change of entropy calculated by Vaspkit[4].

S-VII-2: MoS_2 (100) slab models.

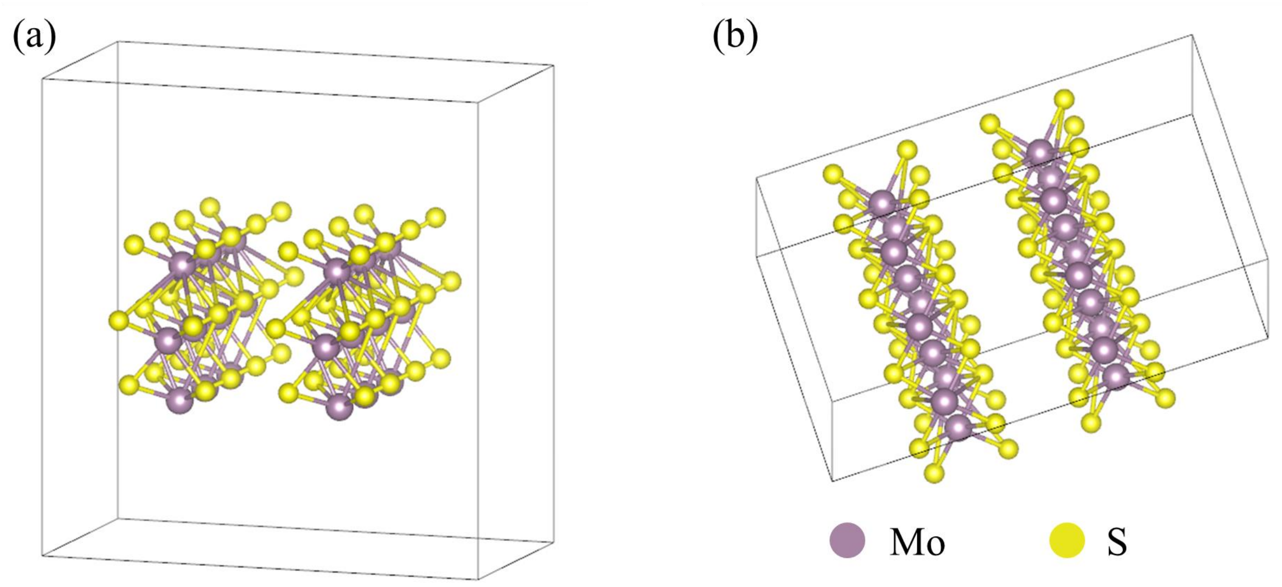

Figure S15. (a) and (b): Different views of the MoS_2 (100) slab models. Light purple and yellow spheres represent Mo and S atoms, respectively. Note that during optimization, the bottom four atomic layers are fixed to mimic the bulk.

S-VII-3: The differential charge density of adsorbed NO_2^- of 2H-MoS_2 and 1T-MoS_2 with different iso-surface value.

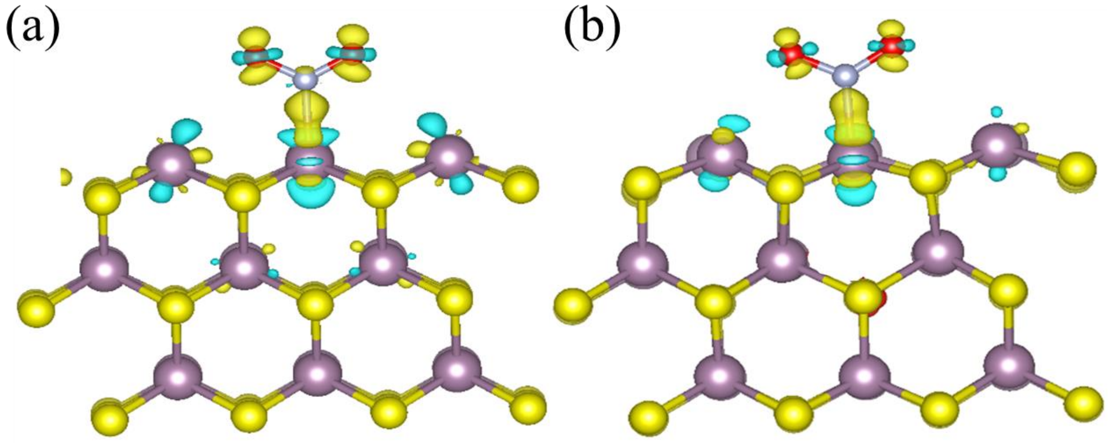

Figure S16. The differential charge density of adsorbed NO_2^- of (a) 2H-MoS_2 and (b) 1T-MoS_2 with the iso-surface value = 0.01 e Å^{-3}.

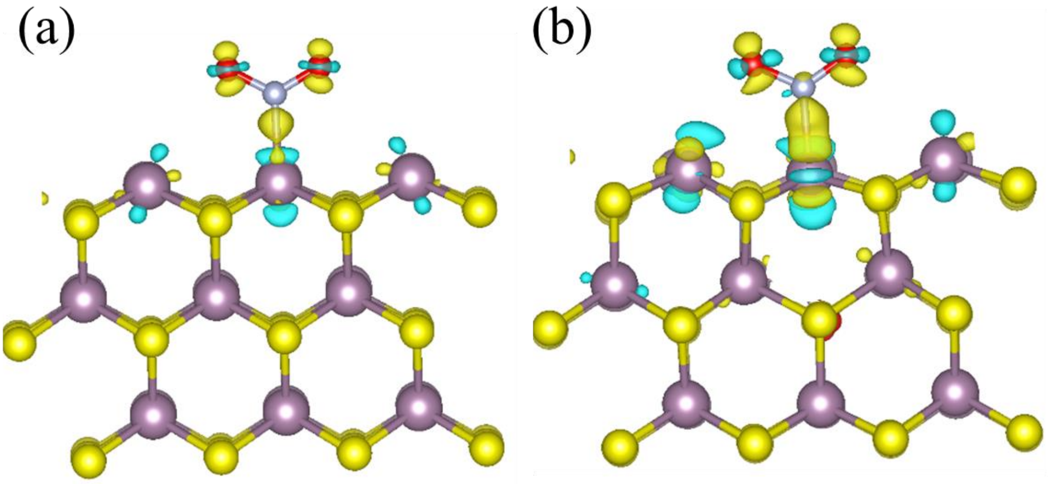

Figure S17. The differential charge density of adsorbed NO_2^- of (a) 2H-MoS_2 and (b) 1T-MoS_2 with the iso-surface value = 0.015 e Å^{-3}.

S-VII-4: PDOS plots of 2H-MoS_2 and 1T-MoS_2.

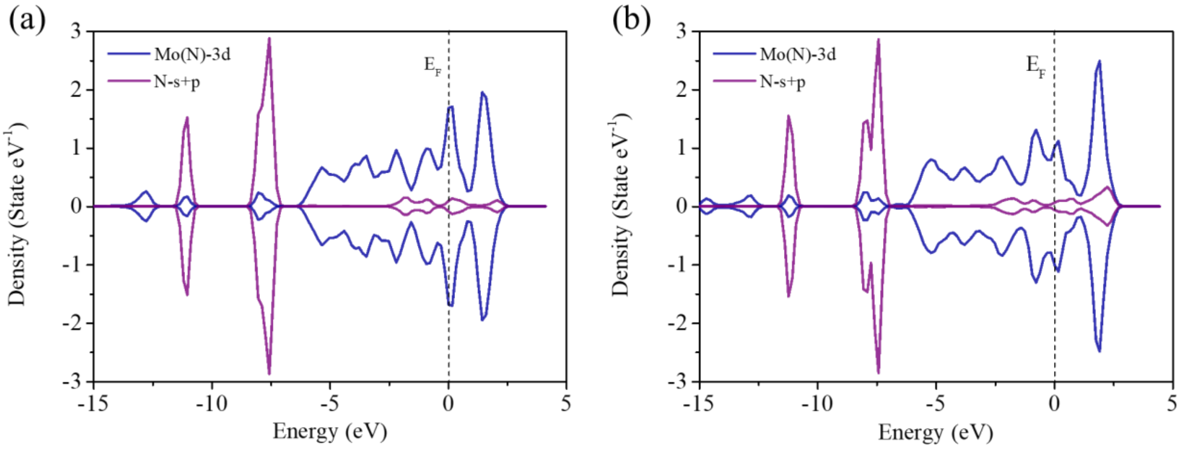

Figure S18. The partial densities of states (PDOS) plots of (a) 2H-MoS_2 and (b) 1T-MoS_2.

S-VII-5: Selected adsorption sites for NO_2^- and H atoms on MoS_2 (100).

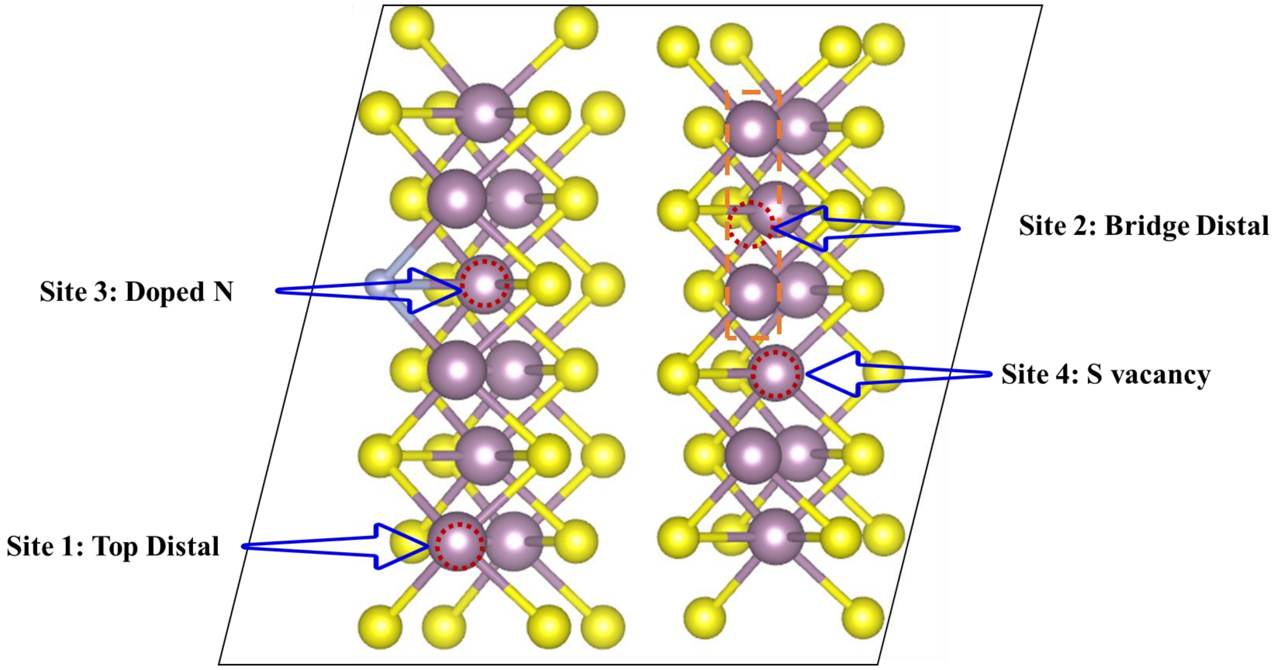

Figure S19. Considered adsorption sites for NO_2^- and H atoms on MoS_2 (100): top Mo distal, Mo-Mo bridge distal, Mo with N-doped sites, and Mo with S vacancies sites.

S-VII-6: Free energy calculation of N-MoS_2 for N_2 adsorption and VGs for NO_2^- adsorption.

Table S3. Free energy comparison of various adsorption sites.

Adsorption Sites	N_2 Adsorption Energy (eV)	NO_2^- Adsorption Energy (eV)
top Mo distal	0.07	-1.12
Mo-Mo bridge distal	0.81	-0.92
Mo with N-doped sites	1.26	0.26
Mo with S vacancies sites	1.07	-0.65
VGs	\	1.54

The free energies for N_2 adsorption are calculated in Section S-VIII-6. Compared with NO_2^- adsorption, the free energies for N_2 are too high (0.07 ~ 1.26 eV) to adsorb N_2 spontaneously, hence, it is a sensible strategy that converting N_2 to NO_2^- realized by plasma.

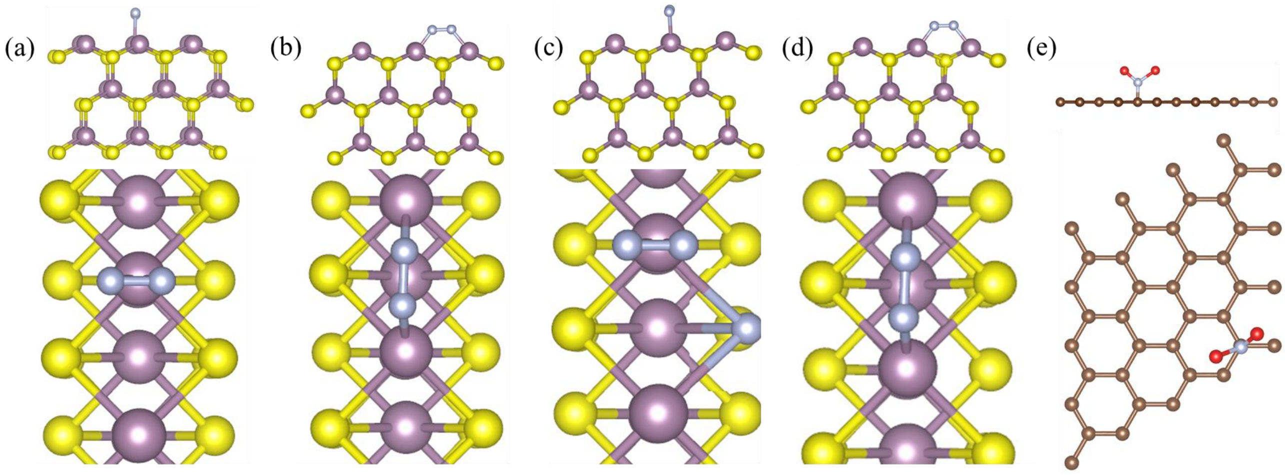

Figure S20. Top and front view of various adsorption sites of N-MoS_2 for N_2 Adsorption: (a) top Mo distal, (b) Mo-Mo bridge distal, (c) Mo with N-doped sites, (d) Mo with S vacancies sites, and (e) VGs for NO_2^- Adsorption.

S-VII-7: Gibbs free energy changes of various eNO_2^-RR pathways.

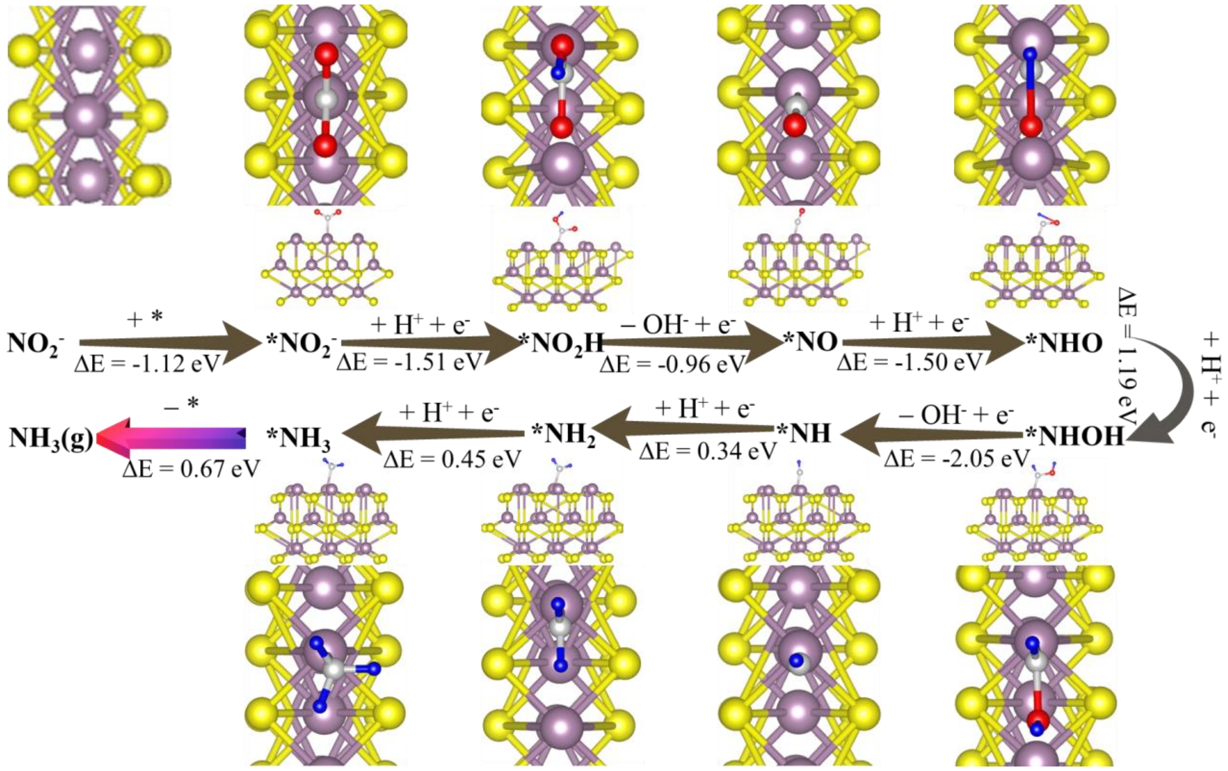

Figure S21. Top and front view of the N-MoS_2 with eNO_2^-RR intermediates adsorbed on the top Mo distal. The Gibbs free energy changes are marked.

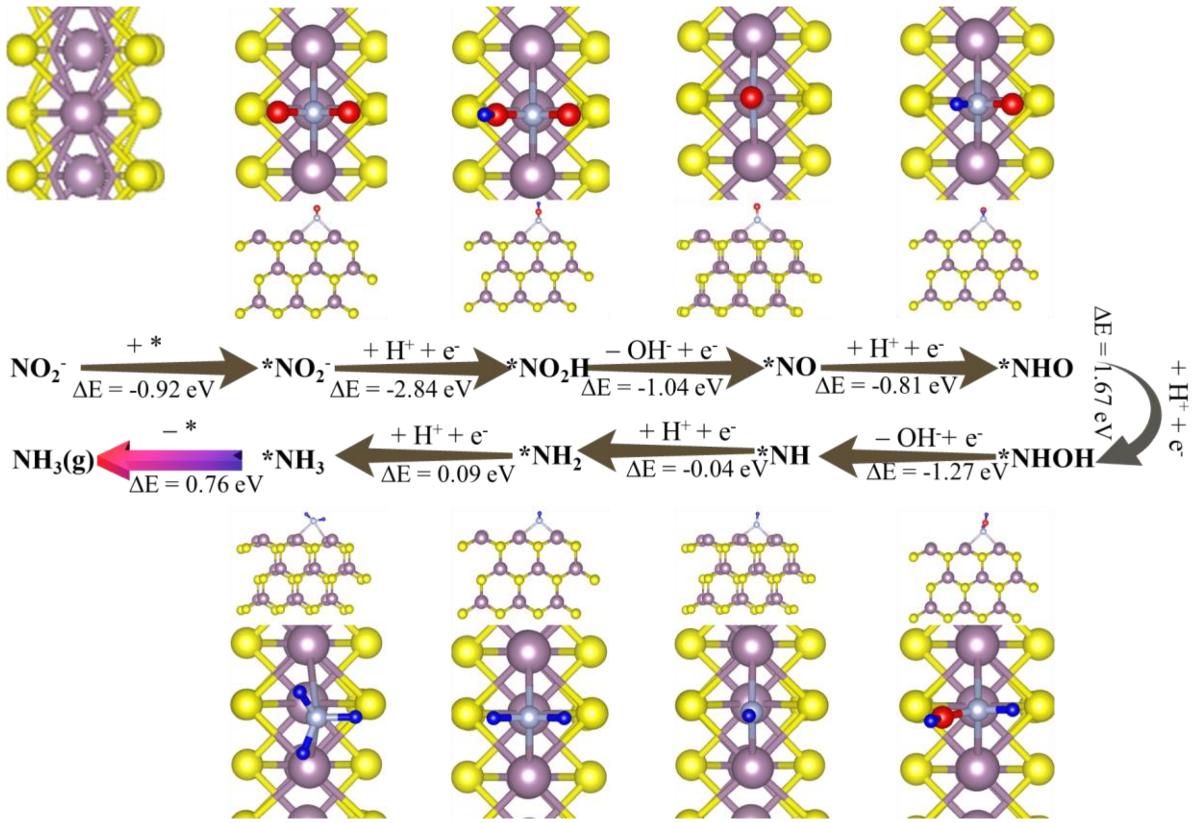

Figure S22. Top and front view of the N-MoS_2 with eNO_2^-RR intermediates adsorbed on the Mo-Mo bridge distal. The Gibbs free energy changes are marked.

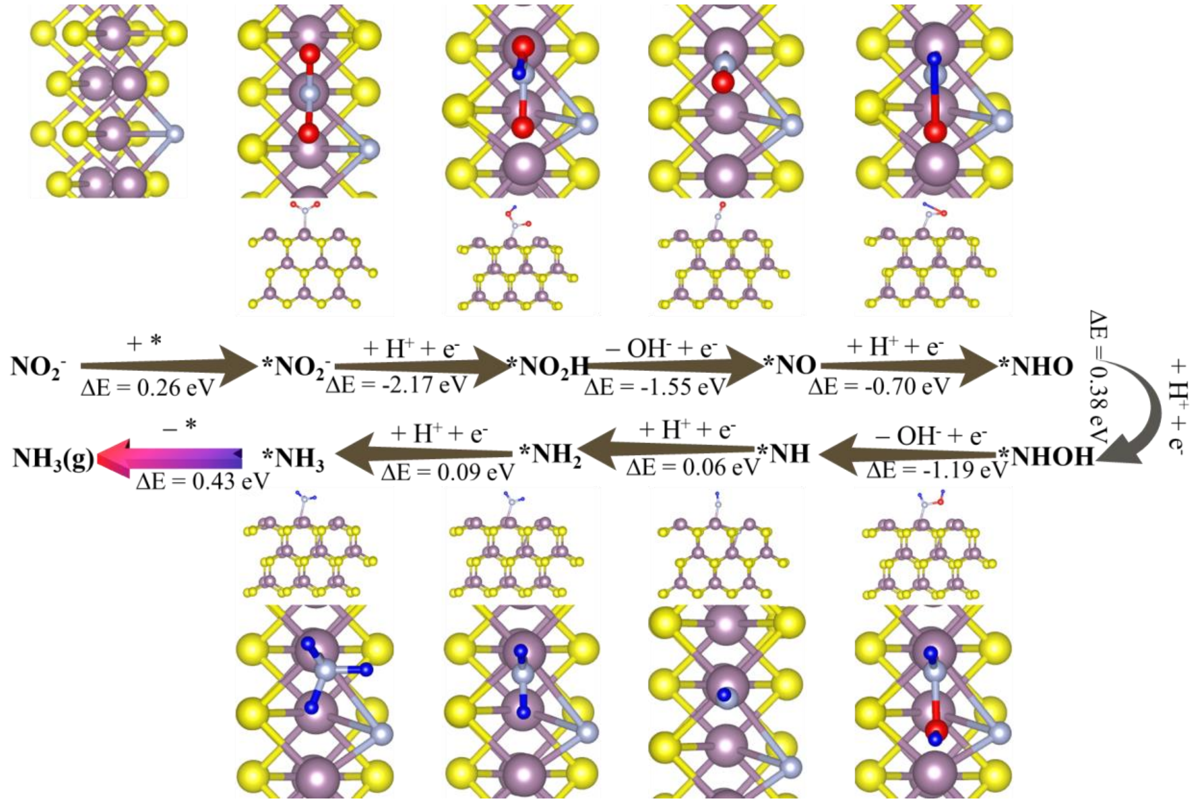

Figure S23. Top and front view of the N-MoS_2 with eNO_2^-RR intermediates adsorbed on the Mo with N-doped sites. The Gibbs free energy changes are marked.

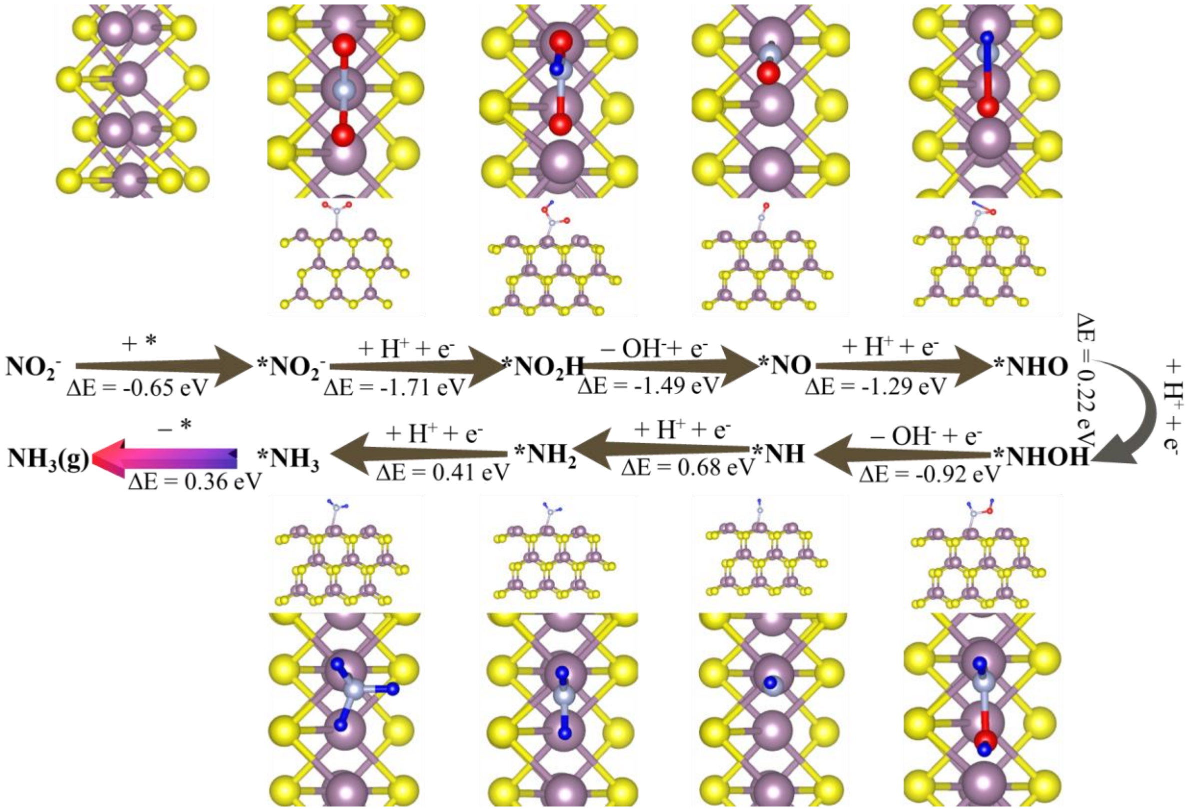

Figure S24. Top and front view of the N-MoS$_2$ with eNO$_2^-$RR intermediates adsorbed on the Mo with S vacancies sites. The Gibbs free energy changes are marked.

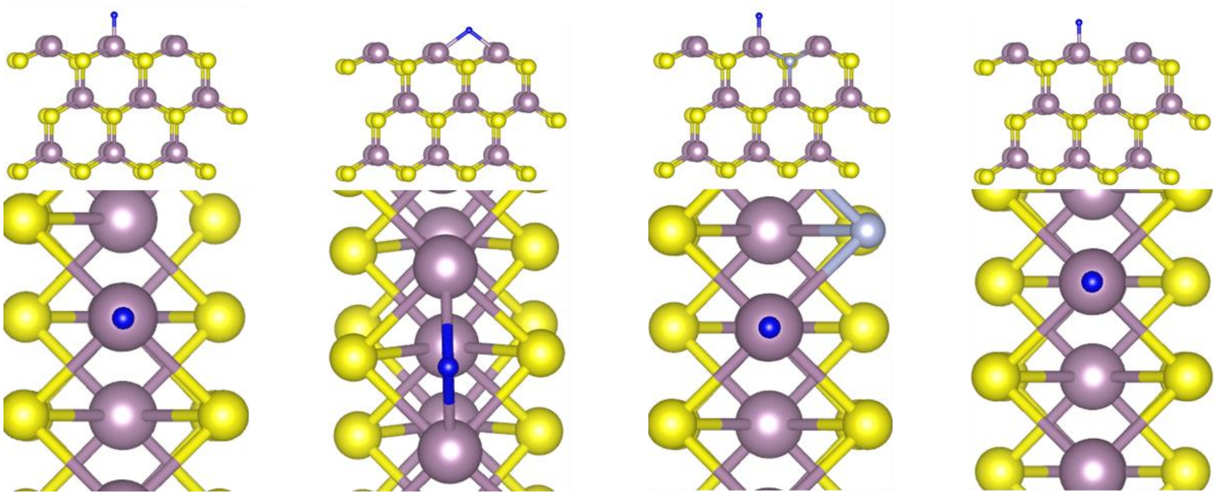

Figure S25. Top and front view of the N-MoS$_2$ with H$^+$ adsorbed on the top Mo distal, Mo-Mo bridge distal, Mo with N-doped sites, and Mo with S vacancies sites.

S-VII-8: HER and eNO_2^-RR Gibbs free energy diagrams of 1T-MoS_2 and 2H-MoS_2

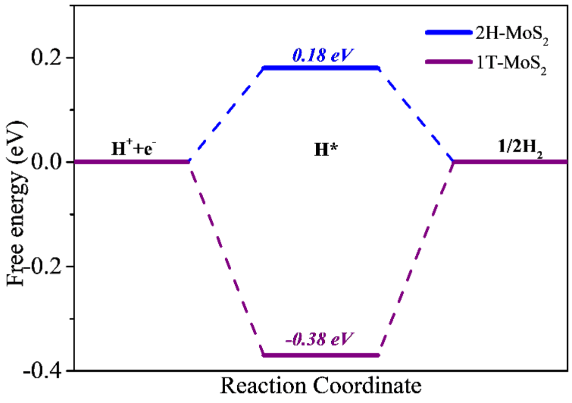

Figure S26. HER Gibbs free energy diagrams of 1T-MoS_2 and 2H-MoS_2.

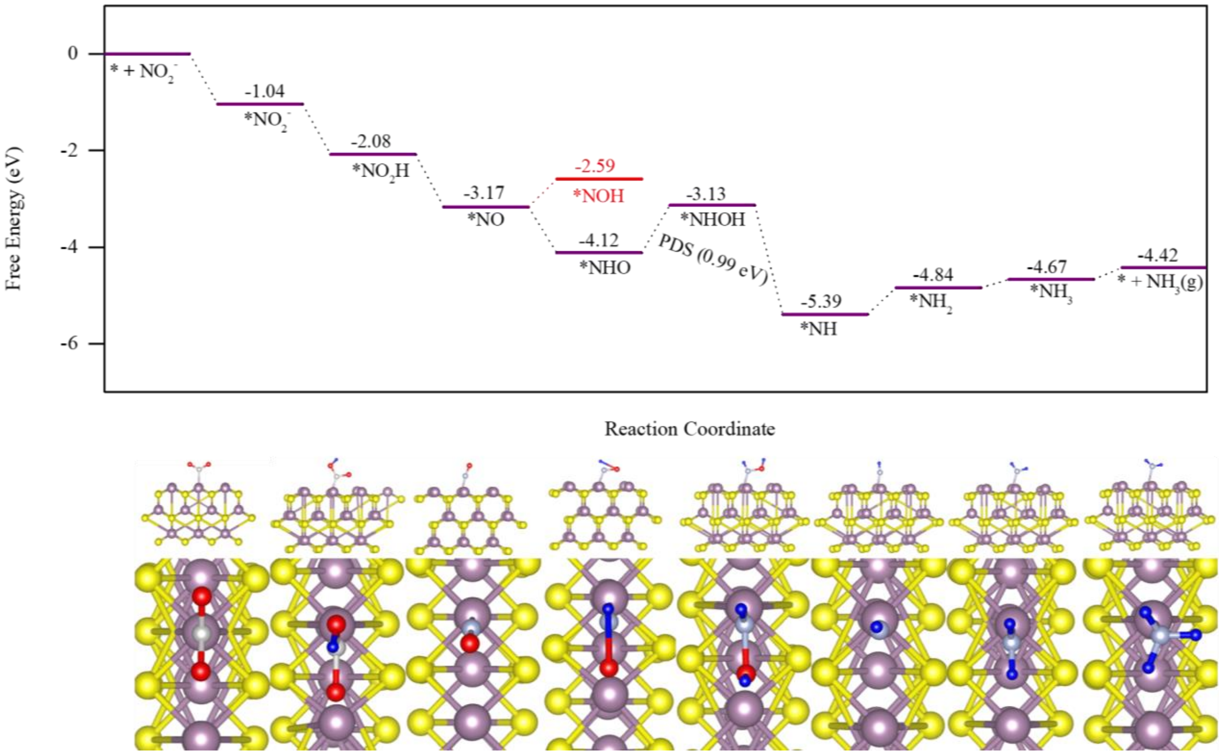

Figure S27. Gibbs free energy diagrams of 1T-MoS_2 and corresponding top and front view of adsorbed eNO_2^-RR intermediates.

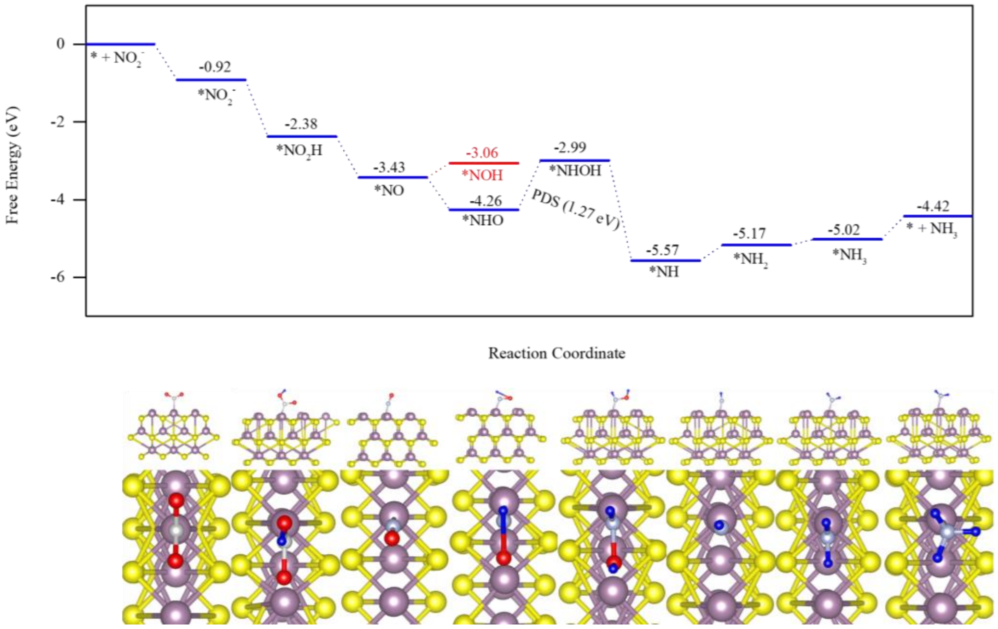

Figure S28. Gibbs free energy diagrams of 2H-MoS_2 and corresponding top and front view of adsorbed eNO_2^-RR intermediates.

In Figure S27-28, the lower free energy of 1T-MoS_2 for PDS (0.99 eV) than 2H-MoS_2 (1.27 eV) indicates the lower energy barrier for the overall reaction. When the electrode potential of PDS is applied, the adsorption energy of NO_2^- for 1T-MoS_2 ($\Delta G_{1T}^{*NO_2^-}$) = -0.05 eV is also more negative than the adsorption energy of NO_2^- for 2H-MoS_2 ($\Delta G_{2H}^{*NO_2^-}$) = 0.35 eV, indicating the preference of NO_2^- for 1T-MoS_2. However, the metallic 1T-MoS_2 also bring the more active HER, which need to be restrained through some reliable approach.

S-VIII: Energy consumption comparison of plasma NO_x generation and eNO_2^-RR.

Table S4. Comparison of overall eNO_2^-RR performance under various applied voltage of plasma at a cell potential of -0.33 V vs RHE.

Power (W)	NO_x (ppm): before passing solution		NO_x (ppm) - after passing solution	Current density (mA cm^{-2})	NH_3 production rate (mg h^{-1})	Energy consumption (MJ mol_{NH3}^{-1})	
	NO	NO_2				Plasma	Electrocatalysis
97	5805	609	~5600	~80	4.7	2.26	1.44
103	8394	1145	~7000	~83	5.0	1.62	1.40
139	11078	1757	~9700	~82	5.4	1.62	1.29
162	13556	2619	~11400	~85	5.8	1.50	1.24
176	15334	3327	~12100	~85	6.2	1.41	1.16

Table S5. Comparison of eNO_2^-RR performance under various potentials of electrocatalysis at the power of 176 W.

Potential (V vs RHE)	Average current density (mA cm^{-2})	NH_3 production rate (mg h^{-1})	Energy consumption (MJ mol_{NH3}^{-1})
-0.13	~25	2.1	0.99
-0.23	~45	3.3	1.15
-0.33	~80	4.7	1.44
-0.43	~70	4.1	1.85
-0.53	~120	5.4	1.95

The detailed methods were given in Section S-I-4: Energy consumption calculation.

S-IX: Summary of reported ammonia synthesis in conventional eNRR and plasma tandem-electrocatalytic systems.

Table S6. Comparison of NRR performance with different MoS_2-based catalysts

Cathode	Conditions		NH_3 production rate (mg h^{-1})	FE (%)	Cell potential (V vs RHE)	Ref.
	Electrolyte	Feed gas				
N-MoS_2/VGs	0.1 M KOH	Air	7.3	38.1	-0.53	This work
1T'''-MoS_2	0.1 M Na_2SO_4	N_2	9.09×10^{-3}	13.6	-0.3	[5]
Fe-MoS_2	0.1 M KOH	N_2	12.5×10^{-3}	10.8	-0.1	[6]
SV-1T-MoS_2@MoO_3	0.05 M H_2SO_4	N_2	116×10^{-3}	18.9	-0.2	[7]
MoS_2@$Fe(OH)_3$	0.1 M Na_2SO_4	N_2	25.9×10^{-3}	2.76	-0.45	[8]
CoS_2/MoS_2	1 M K_2SO_4	N_2	38.61×10^{-3}	34.66	-0.25	[9]
MoS_2@ZIF-71	0.1 M Na_2SO_4	N_2	56.69×10^{-3}	30.91	-0.2	[10]
$Cu_{2-x}S$/MoS_2	0.1 M Na_2SO_4	N_2	22.1×10^{-3}	6.06	-0.5	[11]
FeS@MoS_2/CFC	0.1 M Na_2SO_4	N_2	8.45×10^{-3}	2.96	-0.5	[12]
2H-MoS_2	\	N_2	5.3×10^{-3}	0.023	-1.25	[13]
1T-MoS_2/BTAB/PPy/GO	0.1 M KOH	N_2	13.60×10^{-3}	1.96	-0.49	[14]
CoS_2/MoS_2	0.1 M Li_2SO_4	N_2	54.7×10^{-3}	20.8	-0.6	[15]
FeS_2−MoS_2	0.1 M KOH	N_2	43.45×10^{-3}	4.6	-0.5	[16]
MoS_2@TiO_2	0.1 M Na_2SO_4	N_2	24.14×10^{-3}	65.52	-0.2	[17]
MoS_2/C_3N_4	0.1 M $LiClO_4$	N_2	18.5×10^{-3}	17.8	-0.3	[18]
Ru/2H-MoS_2	10 mM HCl	N_2	6.98×10^{-3}	17.6	-0.15	[19]
MoS_2/C_3N_4	0.1 M Na_2SO_4	N_2	19.86×10^{-3}	6.87	-0.5	[20]
MoS_2 NDs/RGO	0.1 M Na_2SO_4	N_2	16.41×10^{-3}	27.93	-0.75	[21]
MoS_2	0.1 M Na_2SO_4	N_2	4.9×10^{-3}	1.17	-0.5	[22]
CNMS	0.1 M Na_2SO_4	N_2	71.07×10^{-3}	21.01	-0.5	[23]
N-MoS_2	0.1 M Na_2SO_4	N_2	69.82×10^{-3}	9.14	-0.3	[24]
V_S-MoS_2	0.1 M Na_2SO_4	N_2	29.55×10^{-3}	4.58	-0.5	[25]
3R-MoS_2	0.1 M Na_2SO_4	N_2	8.8×10^{-3}	1.9	-1.0	[26]
F-MoS_2	0.05 M H_2SO_4	N_2	35.7×10^{-3}	20.6	-0.2	[27]
1T-MoS_2–Ni	0.1 M $LiClO_4$	N_2	63×10^{-3}	27.66	-0.3	[28]
MoS_2 QDs	0.1 M Na_2SO_4	N_2	39.6×10^{-3}	12.9	-0.3	[29]
1T'-MoS_2/TiO_2	0.1 M Na_2SO_4	N_2	29.62×10^{-3}	24.9	-0.75	[30]

Table S7. Compilation of reported ammonia synthesis in the plasma tandem-electrocatalysis system.

Ref.	Plasma	Reactants	Catalyst	Flow rate (L min^{-1})	Role of Plasma	Energy Consumption (MJ mol^{-1})	N_2 Conversion (%)	NH_3 Production Rate (mg h^{-1})	Electrolyte	Comment
This work	GA	Air	N-MoS_2/VGs	9	N_2 oxidation	2.40	1.12	7.3	0.1M KOH	@-0.53V vs RHE
[31]	Spark	Air	Cu nanoparticles	0.03	N_2 activation	\	\	2.4	0.1M KOH	@-0.9V vs RHE
[32]	Rotating Gliding Arc (RGA)	Air	Co-SACs	7	Intermediate production	3.18	1.00	3.0	0.1M KOH	@-0.53V vs RHE
[33]	Dielectric Barrier Discharge (DBD)	Air	Ni_3B@$NiB_{2.74}$	0.04	N_2 oxidation	\	0.68	1.8	0.1M KOH	@-0.3V vs RHE
[34]	DBD	Air	Cu-NW	2	Air activation	15.48	0.02	2.4	0.01M H_2SO_4	@-0.6V vs RHE
[35]	Jet	N_2	TiO_2	1	N_2 dissociation	275.51	\	1.7×10^{-3}	0.01M H_2SO_4	\
[36]	Jet	N_2-H_2O	\	\	Serve as the cathode	\	\	0.44	H_2SO_4	\
[37]	Jet	N_2-He	Pt/C	\	N_2 activation	\	\	0.032	DI	\

S-X: Summary of reported solar powered plasma setups, and solar-driven electrocatalysis.

Table S8. Summary of solar-powered plasma setups.

Plasma type	Application	Results	Ref.
Gliding arc	CO_2 decomposition	net-absorption of solar radiation up to 18%; gas-phase decomposition up to 4.5%.	[38]
DBD	NO_x removal	Solar powered battery voltage up to 24 V and convert to 18 kV;<br>50% of NO_x can be removed at 120 J L^{-1} with a gas flow rate of 2 L min^{-1};<br>Removal efficiency can be increased to 24 J L^{-1} with gas flow rate of 10 L min^{-1}.	[39]
Microwave	CO_2 conversion	Absorption of solar power obtained under CO_2-Ar operation up to ~21%;	[40]
Microwave	CO_2 conversion	The highest conversion efficiency obtained with CO_2-N_2 is ~15.5%, whereas the highest conversion efficiency using CO_2-Ar is ~9.5%.	[41]
DBD	NO_x removal	71% of NO_X can be removed with gas flow rate of 2 L min^{-1};<br>DeNO_x efficiency of 22% is achieved.	[42]

Table S9. Summary of solar-driven electrocatalysis.

Ref.	Photovoltaic	Reaction	Cathode	Electrolyte	Conversion efficiency (%)	Stability
[43]	GaInP/GaAs/Ge	Nitrate reduction	OD-Co	1 M KNO_3	11	95% (24 h)
[44]	$MAPbI_3$	Water splitting	NiFe	1 M NaOH	12.3	80% (2 h)
[45]	$(FAPbI_3)_{1-x}(MAPbBr_3)_x$	Water splitting	CoP	1 M KOH	9.02	70% (16 h)
[46]	CsFAMA triple cation perovskite	CO_2RR	DN-CuO	0.1 M $CsHCO_3$	9.7	100 (6 h)
[47]	$MAPbI_3$	CO_2RR	Au	0.5 M $NaHCO_3$	6.5	100% (18 h)
[48]	$FA_{0.80}MA_{0.15}Cs_{0.05}PbI_{2.55}Br_{0.45}$	Water splitting	CoP/Sn	1 M NaOH	8.54	90% (3 h)
[49]	$MAPbI_3$	Water splitting	CoP	1 M KOH	6.7	70% (700 s)
[50]	$(Cs_{0.19}FA_{0.81}Pb(Br_{0.13}I_{0.87})_3Si$	Water splitting	TiC/Pt	1 M NaOH	18.7	96% (2 h)
[51]	$MAPb(I_{0.85}Br_{0.15})_3$-Si	Water splitting	Ni_4Mo	1 M NaOH	17.52	100 (300 s)
[51]	$CH_3NH_3PbI_{3-x}Cl_x$	Water splitting	NF-8/CFP	1 M KOH	9.7	90% (500 s)
[52]	$MAPbI_3$−organic PV	Water splitting	NrGO/ NCNT	1 M KOH	2.3	100% (50 min)

References

[1] J. Furthmüller, G. Kresse, *Physical Review B* **1996**, *54*, 11169-11186.

[2] K. Burke, M. Ernzerhof, J. P. Perdew, *Phys. Rev. Lett.* **1997**, *78*, 1396.

[3] V. Wang, N. Xu, J. Liu, G. Tang, W. Geng, *Comput. Phys. Commun.* **2021**, *267*, 108033.

[4] G. Lin, Q. Ju, X. Guo, W. Zhao, S. Adimi, J. Ye, Q. Bi, J. Wang, M. Yang, F. Huang, *Adv. Mater.* **2021**, *33*, 2007509.

[5] X. Zhao, X. Zhang, Z. Xue, W. Chen, Z. Zhou, T. Mu, *J. Mater. Chem. A* **2019**, *7*, 27417-27422.

[6] X. Zi, J. Wan, X. Yang, W. Tian, H. Zhang, Y. Wang, *Applied Catalysis B: Environmental* **2021**, *286*, 119870.

[7] X. Xu, X. Liu, J. Zhao, D. Wu, Y. Du, T. Yan, N. Zhang, X. Ren, Q. Wei, *J. Colloid Interf. Sci.* **2022**, *606*, 1374-1379.

[8] C. Wang, M. Yang, X. Wang, H. Ma, Y. Tian, H. Pang, L. Tan, K. Gao, *J. Colloid Interf. Sci.* **2022**, *609*, 815-824.

[9] J. Duan, D. Shao, X. He, Y. Lu, W. Wang, *Colloids and Surfaces A: Physicochemical and Engineering Aspects* **2021**, *619*, 126529.

[10] T. Jiang, L. Li, L. Li, Y. Liu, D. Zhang, D. Zhang, H. Li, B. Mao, W. Shi, *Chem. Eng. J.* **2021**, *426*, 130650.

[11] Y. Guo, Z. Yao, B. J. J. Timmer, X. Sheng, L. Fan, Y. Li, F. Zhang, L. Sun, *Nano Energy* **2019**, *62*, 282-288.

[12] I. Matanovic, K. Leung, S. J. Percival, J. E. Park, P. Lu, P. Atanassov, S. S. Chou, *Applied Materials Today* **2020**, *21*, 100812.

[13] H. Mao, Y. Fu, H. Yang, Z. Deng, Y. Sun, D. Liu, Q. Wu, T. Ma, X. Song, *ACS Appl. Mater. Inter.* **2020**, *12*, 25189-25199.

[14] G. Yang, L. Zhao, G. Huang, Z. Liu, S. Yu, K. Wang, S. Yuan, Q. Sun, X. Li, N. Li, *ACS Appl. Mater. Inter.* **2021**, *13*, 21474-21481.

[15] M. Yang, Z. Jin, C. Wang, X. Cao, X. Wang, H. Ma, H. Pang, L. Tan, G. Yang, *ACS Appl. Mater. Inter.* **2021**, *13*, 55040-55050.

[16] W. Ye, M. Arif, X. Fang, M. A. Mushtaq, X. Chen, D. Yan, *ACS Appl. Mater. Inter.* **2019**, *11*, 28809-28817.

[17] K. Chu, Y. Liu, Y. Li, Y. Guo, Y. Tian, *ACS Appl. Mater. Inter.* **2020**, *12*, 7081-7090.

[18] B. H. R. Suryanto, D. Wang, L. M. Azofra, M. Harb, L. Cavallo, R. Jalili, D. R. G. Mitchell, M. Chatti, D. R. MacFarlane, *ACS Energy Letters* **2019**, *4*, 430-435.

[19] Z. Zhao, S. Luo, P. Ma, Y. Luo, W. Wu, Y. Long, J. Ma, *ACS Sustain. Chem. Eng.* **2020**, *8*, 8814-8822.

[20] Y. Liu, W. Wang, S. Zhang, W. Li, G. Wang, Y. Zhang, M. Han, H. Zhang, *ACS Sustain. Chem. Eng.* **2020**, *8*, 2320-2326.

[21] L. Zhang, X. Ji, X. Ren, Y. Ma, X. Shi, Z. Tian, A. M. Asiri, L. Chen, B. Tang, X. Sun, *Adv. Mater.* **2018**, *30*, 1800191.

[22] R. Liu, T. Guo, H. Fei, Z. Wu, D. Wang, F. Liu, *Advanced Science* **2022**, *9*, 2103583.

[23] L. Zeng, S. Chen, J. van der Zalm, X. Li, A. Chen, *Chem. Commun.* **2019**, *55*, 7386-7389.

[24] B. Liu, C. Ma, D. Liu, S. Yan, *ChemElectroChem* **2021**, *8*, 3030-3039.

[25] B. Fang, J. Yao, X. Zhang, L. Ma, Y. Ye, J. Tang, G. Zou, J. Zhang, L. Jiang, Y. Sun, *New J. Chem.* **2021**, *45*, 2488-2495.

[26] J. Liang, S. Ma, J. Li, Y. Wang, J. Wu, Q. Zhang, Z. Liu, Z. Yang, K. Qu, W. Cai, *J. Mater. Chem. A* **2020**, *8*, 10426-10432.
[27] S. B. Patil, H. Chou, Y. Chen, S. Hsieh, C. Chen, C. Chang, S. Li, Y. Lee, Y. Lin, H. Li, Y. J. Chang, Y. Lai, D. Wang, *J. Mater. Chem. A* **2021**, *9*, 1230-1239.
[28] Y. Luo, P. Shen, X. Li, Y. Guo, K. Chu, *Chem. Commun.* **2021**, *57*, 9930-9933.
[29] X. Xu, Y. Wang, X. Chen, X. Qian, Z. Liang, H. Cui, J. Tian, M. Shao, *EcoMat* **2021**, *3*, e12122.
[30] Y. Ren, C. Yu, L. Wang, X. Tan, Z. Wang, Q. Wei, Y. Zhang, J. Qiu, *J. Am. Chem. Soc.* **2022**.
[31] A. Wu, J. Yang, B. Xu, X. Wu, Y. Wang, X. Lv, Y. Ma, A. Xu, J. Zheng, Q. Tan, Y. Peng, Z. Qi, H. Qi, J. Li, Y. Wang, J. Harding, X. Tu, A. Wang, J. Yan, X. Li, *Applied Catalysis B: Environmental* **2021**, *299*, 120667.
[32] L. Li, C. Tang, X. Cui, Y. Zheng, X. Wang, H. Xu, S. Zhang, T. Shao, K. Davey, S. Qiao, *Angewandte Chemie International Edition* **2021**, *60*, 14131-14137.
[33] J. Sun, D. Alam, R. Daiyan, H. Masood, T. Zhang, R. Zhou, P. J. Cullen, E. C. Lovell, A. R. Jalili, R. Amal, *Energ. Environ. Sci.* **2021**, *14*, 865-872.
[34] P. Lamichhane, B. C. Adhikari, L. N. Nguyen, R. Paneru, B. Ghimire, S. Mumtaz, J. S. Lim, Y. J. Hong, E. H. Choi, *Plasma Sources Science and Technology* **2020**, *29*, 45026.
[35] R. Hawtof, S. Ghosh, E. Guarr, C. Xu, S. R. Mohan, J. N. Renner, *Science Advances*, *5*, t5778.
[36] S. Kumari, S. Pishgar, M. E. Schwarting, W. F. Paxton, J. M. Spurgeon, *Chem. Commun.* **2018**, *54*, 13347-13350.
[37] D. Nagassou, S. Mohsenian, S. Bhatta, R. Elahi, J. P. Trelles, *Sol. Energy* **2019**, *180*, 678-689.
[38] S. Das, P. Manaswini, P. Kumari, S. Mohapatro, B. Rajanikanth, *International Journal on Control System & Instrumentation* **2010**.
[39] S. Mohsenian, D. Nagassou, R. Elahi, P. Yu, M. Nallar, H. Wong, J. P. Trelles, *Journal of CO2 Utilization* **2019**, *34*, 725-732.
[40] S. Mohsenian, D. Nagassou, S. Bhatta, R. Elahi, J. P. Trelles, *Plasma Sources Science and Technology* **2019**, *28*, 65001.
[41] Mohapatro, Sankarsan, Rajanikanth, S. B., *IEEE Transactions on Dielectrics & Electrical Insulation* **2011**, *18*, 1821-1828.
[42] N. C. Kani, J. A. Gauthier, A. Prajapati, J. Edgington, I. Bordawekar, W. Shields, M. Shields, L. C. Seitz, A. R. Singh, M. R. Singh, *Energ. Environ. Sci.* **2021**, *14*, 6349-6359.
[43] J. Luo, J. Im, M. T. Mayer, M. Schreier, M. K. Nazeeruddin, N. Park, S. D. Tilley, H. J. Fan, M. Grätzel, *Science* **2014**, *345*, 1593-1596.
[44] J. Luo, D. A. Vermaas, D. Bi, A. Hagfeldt, W. A. Smith, M. Grätzel, *Adv. Energy Mater.* **2016**, *6*, 1600100.
[45] T. N. Huan, C. D. A. Dalla, S. Lamaison, D. Karapinar, L. Lutz, N. Menguy, M. Foldyna, S. Turren-Cruz, A. Hagfeldt, F. Bella, M. Fontecave, V. Mougel, *Proceedings of the National Academy of Sciences* **2019**, *116*, 9735-9740.
[46] M. Schreier, L. Curvat, F. Giordano, L. Steier, A. Abate, S. M. Zakeeruddin, J. Luo, M. T. Mayer, M. Grätzel, *Nat. Commun.* **2015**, *6*, 7326.
[47] H. Chen, M. Zhang, T. Tran-Phu, R. Bo, L. Shi, I. Di Bernardo, J. Bing, J. Pan, S. Singh, J. Lipton-Duffin, T. Wu, R. Amal, S. Huang, A. W. Y. Ho-Baillie, A. Tricoli, *Adv. Funct. Mater.* **2021**, *31*, 2008245.
[48] J. Liang, X. Han, Y. Qiu, Q. Fang, B. Zhang, W. Wang, J. Zhang, P. M. Ajayan, J. Lou, *ACS Nano* **2020**, *14*, 5426-5434.

[49] J. Gao, F. Sahli, C. Liu, D. Ren, X. Guo, J. Werner, Q. Jeangros, S. M. Zakeeruddin, C. Ballif, M. Grätzel, J. Luo, *Joule* **2019**, *3*, 2930-2941.

[50] H. Park, I. J. Park, M. G. Lee, K. C. Kwon, S. Hong, D. H. Kim, S. A. Lee, T. H. Lee, C. Kim, C. W. Moon, D. Son, G. H. Jung, H. S. Yang, J. R. Lee, J. Lee, N. Park, S. Y. Kim, J. Y. Kim, H. W. Jang, *ACS Appl. Mater. Inter.* **2019**, *11*, 33835-33843.

[51] A. Kumar, D. K. Chaudhary, S. Parvin, S. Bhattacharyya, *J. Mater. Chem. A* **2018**, *6*, 18948-18959.

[52] A. R. Bin, M. Yusoff, J. Jang, *Chem. Commun.* **2016**, *52*, 5824-5827.
